# Oral administration of oat beta-glucan preparations of different molecular weight results in regulation of genes connected with immune response in peripheral blood of rats with LPS-induced enteritis

**DOI:** 10.1007/s00394-018-1838-3

**Published:** 2018-10-04

**Authors:** Katarzyna Błaszczyk, Małgorzata Gajewska, Jacek Wilczak, Dariusz Kamola, Alicja Majewska, Joanna Harasym, Joanna Gromadzka-Ostrowska

**Affiliations:** 1 Department of Dietetics, Faculty of Human Nutrition and Consumer Sciences, Warsaw University of Life Sciences, Nowoursynowska 159c, 02-776 Warsaw, Poland; 2 Biochemistry Division, Department of Physiological Sciences, Faculty of Veterinary Medicine, Warsaw University of Life Sciences, Nowoursynowska 159, 02-787 Warsaw, Poland; 3 Dietetics Division, Department of Physiological Sciences, Faculty of Veterinary Medicine, Warsaw University of Life Sciences, Nowoursynowska 159, 02-787 Warsaw, Poland; 4 BIO-REF@LAB, Department of Biotechnology and Food Analysis, Faculty of Engineering and Economics, Wrocław University of Economics, Komandorska 118/120, 53-345 Wrocław, Poland

**Keywords:** Autophagy, Beta-glucans, Enteritis, Immunomodulation, Transcriptomic profile

## Abstract

**Purpose:**

Beta-glucans are biologically active polysaccharides having antioxidant, immunomodulatory, and antiinflammatory properties. This study investigated the transcriptomic profile in peripheral blood of rats with LPS-induced *enteritis*, which were fed a diet supplemented with high- (G1) and low- (G2) molecular-weight oat beta-glucans.

**Methods:**

Two-color rat gene expression microarrays were applied and the analysis was performed using a common reference design to provide easy means of comparing samples from various experimental conditions against one another. Common reference sample was labeled with cyanine 3 (Cy3) and investigated samples from each experimental group: C-G0 (control group fed semi-synthetic diet), LPS-G0 (LPS-challenged group fed semi-synthetic diet), LPS-G1 (LPS-challenged group fed G1 beta-glucan enriched diet), and LPS-G2 (LPS-challenged group fed G2 beta-glucan enriched diet) were labeled with cyanine 5 (Cy5). Each microarray was performed in quadruplicate. Statistical analysis was performed using one-way ANOVA and Tukey’s HSD post-hoc test (*p* < 0.05). A multiple testing correction was performed using Benjamini and Hochberg False Discovery Rate < 5%. A quantitative real-time RT-PCR was performed to verify the expression of chosen transcripts.

**Results:**

The microarray analyses revealed differentially expressed transcripts between: the LPS-G0 and the control groups: C-G0 (138 genes), the LPS-G1 and LPS-G0 groups (533 genes), and the LPS-G2 and LPS-G0 groups (97 genes). Several differentially expressed genes in the beta-glucan-supplemented groups encoded proteins belonging to TLR and NLR signaling pathways, as well as prostaglandin synthesis and regulation pathways. Both beta-glucans up-regulated the expression of *Atg10*, which belongs to the family of autophagy-related genes, suggesting a possible link between autophagy induction and beta-glucan supplementation.

**Conclusion:**

The changes in gene expression observed in the peripheral blood indicate that oat beta-glucans exerted a protective effect in rats with an induced inflammatory state caused by LPS challenge. The greater number of differentially expressed genes was observed in group supplemented with G1 beta-glucan, pointing at the differences in the mode of action of high- and low-molecular-weight beta-glucans in the organism.

**Electronic supplementary material:**

The online version of this article (10.1007/s00394-018-1838-3) contains supplementary material, which is available to authorized users.

## Introduction

Polysaccharides are known to play a crucial role in potentiating immunity, and they are predicted to provide a wide area for the discovery of new types of safe and tolerable immune adjuvants. Among numerous investigated polysaccharides, beta-glucans belong to the most promising in terms of their potential therapeutic properties [1]. Beta-glucans are a group of biologically active natural compounds and represent highly conserved structural components of cell walls in fungi, yeast, seaweed, and cereals. In general, beta-glucan is the chemical name of a polymer of beta-glucose, and exists as a homopolymer of glucose having an unbranched chain with (1 → 3)-beta-d-glycosidic linkages, mixed-linked (1 → 3),(1 → 4)-beta-d-glycosidic linkages, or a branched polymer with side chains joined by (1 → 6)-beta-d glycosidic linkages. The mechanisms of beta-glucan action include a reduction in nutrient absorption and an improvement in intestinal content viscosity. Furthermore, beta-glucans can be a potential source for fermentation by the microbes present in the small intestine [2] and may produce a prebiotic effect [3]. Such an effect was also observed in our studies, where we demonstrated that the potential prebiotic properties of beta-glucans were mostly connected with the stimulation of short-chain fatty acids production by intestinal microflora, rather than changes in the number of lactic acid bacteria [4].

Extensive research conducted in recent decades has shown that beta-glucans of different origin are able to significantly stimulate several types of immune reactions oriented against microbes, toxic factors, cancer, and other factors [5, 6]. Beta-glucans are able to stimulate the immune response due to their ability to bind to surface receptors of immune cells, i.e., monocytes, granulocytes, and NK cells, and to their ability to activate and regulate humoral as well as cell-mediated immunity. It has been established that beta-glucans may act via three distinct immunostimulating mechanisms, modulating the activity of: macrophages, T lymphocytes, and the complement system. Due to these properties beta-glucans are able to regulate innate immune response as well as to enhance adaptive immunity by stimulating the activity of T lymphocytes. Studies using animal models have demonstrated that dietary supplementation with beta-glucans enhances the immune system by activating T cells expressing CD8 and TCR1 surface molecules [7].

Our previous studies showed the beneficial effects of oat beta-glucans on the health status of rats with *enteritis* induced by LPS injection [4, 8–10], and other studies have shown that the molecular weight of oat beta-glucans determines the mode of action of these compounds in the body [11]. Our observations revealed that high-molecular-weight beta-glucan was more effective in decreasing oxidative stress in animals with LPS-induced *enteritis* in colon tissue, as well as in the stomach, liver, or spleen [4, 8–10]. On the other hand, dietary supplementation with low-molecular-weight beta-glucan resulted in an improvement in the condition (morphology) of colon tissue in healthy control rats and animals with LPS-induced *enteritis* [4].

Therefore, in the present research, we aimed to investigate whether dietary supplementation with high- and low-molecular-weight beta-glucans (G1 and G2, respectively) may also have a systemic effect in rats challenged with LPS, which would manifest in, among other things, changes in gene expression observed in peripheral blood. To our knowledge, this is the first study investigating the potential genomic effect of dietary beta-glucans and intravenously administered LPS in blood of rats with LPS-induced *enteritis*.

## Materials and methods

### Characterization of beta-glucan preparations from oat

Two fractions of oat beta-glucans: high molecular weight (G1, 2,180,000 g/mol) and low molecular weight (G2, 70,000 g/mol) were used in this experiment. High-molecular-weight oat beta-glucan fraction was obtained from Futurum Ltd. (Częstochowa, Poland) and produced on the basis of a patented procedure [12]. Low-molecular-weight oat beta-glucan fraction was manufactured accordingly with a method previously published by our research group [13]. Methods of extraction and characterisation of beta-glucans have been described in details in our earlier report, regarding the effect of these compounds on the antioxidative and antiinflammatory status of rats [8]. Briefly, oat beta-glucans of high and low molecular weights were isolated from beta-glucan enriched oat fiber (20%, Microstructure, Poland) by water extraction in alkaline conditions (pH 8, 1 M NaOH), followed by deproteination, precipitation with ethanol addition, and drying (80 °C, 24 h) [12, 13]. The content of oat beta-glucans in samples was evaluated with lichenase hydrolysis method and analyzed spectrophotometrically according to the AOAC 995.16 method, which was modified to determine the high beta-glucan content (cellulase addition, Megazyme, Ireland), as described in our previous paper [13]. Molecular weight was determined by HPLC-SEC with commercially available beta-glucans’ standards (Megazyme, Ireland) [13]. All samples were run in triplicates and the results were presented as mean values.

### Animals and treatment

The study was performed on 8-week-old male Sprague–Dawley rats (*n* = 72) obtained from Charles River Laboratories (Sulzfeld, Germany). All used protocols had the approval of the IIIrd local Ethical Committee in Warsaw, Poland, and respected the 3 R rules. Animals were separated and kept in individual polyurethane cages for a period of 6 weeks with food and water available ad libitum. The environment was controlled, with a 12 h light/dark cycle, a room temperature of 22 ± 0.5 °C, and a relative humidity of 50%. Body weight was recorded at regular intervals (once a week) and the feed consumption was measured every 2 days, from the end of the 2 week test period.

At the beginning of experiment, animals were divided into two major groups: a control group (group C, *n* = 36) and an experimental group (group LPS, *n* = 36). Animals from the C group were injected (through the tail vein) with aqueous sodium chloride solution (0.9%) administered twice at intervals of 7 days, while rats in the experimental group were injected with *E. coli* lipopolysaccharides (LPS; 10 mg per kg of body weight; administered twice at intervals of 7 days), to induce *enteritis*. The previous studies have shown that systemic administration of LPS to animals induces small intestine inflammation [4, 14].

Rats in control and LPS-challenged groups were further divided into six subgroups (*n* = 12 rats, each) according to the experimental diets that they received in the course of the 6-week experiment. Animals were fed semi-synthetic diet (control: G0 diet) formulated with respect to the nutritional requirements for laboratory animals (National Research Council 1995) or semi-synthetic diet supplemented with 1% (w/w) oat beta-glucans (G1 and G2 diets). Thus, the following experimental groups of rats were created: C-G0 and LPS-G0 (control and *enteritis* individuals, fed control diet), C-G1 and LPS-G1 (control and *enteritis* individuals, fed diet with high-molecular-weight oat beta-glucan), and C-G2 and LPS-G2 (control and *enteritis* individuals, fed diet with low-molecular-weight oat beta-glucan). Semi-synthetic diet was implemented to avoid the possible influence of beta-glucans present in commercially available diets. Composition of the diets (micronutrients, macronutrients, and vitamins mixtures) was described elsewhere [4]. Rats body weight gain and feed consumption were measured during the experimental period, and the results have been published in our previous report [8].

### Microarrays

#### RNA isolation, validation, labeling, and hybridization

At the end of the 6-week feeding period, after 12 h of fasting, blood samples were taken by the cardiac puncture under isoflurane anesthesia and collected in RNeasy Protect Animal Blood Tubes (Qiagen, USA). Total RNA from peripheral blood was isolated using a RNeasy Protect Animal Blood Kit (Qiagen, USA). Isolated RNA samples were dissolved in 30 µl of REB Buffer from the test kit. Next, the RNA quantity was measured spectrophotometrically using a NanoDrop spectrophotometer (NanoDrop Technologies, USA). The analysis of the final RNA quality and integrity was performed with a BioAnalyzer (Agilent, USA). To ensure optimal data quality, only RNA samples with RIN number ≥ 7.5 were included in the analysis.

**Fig. 1:**
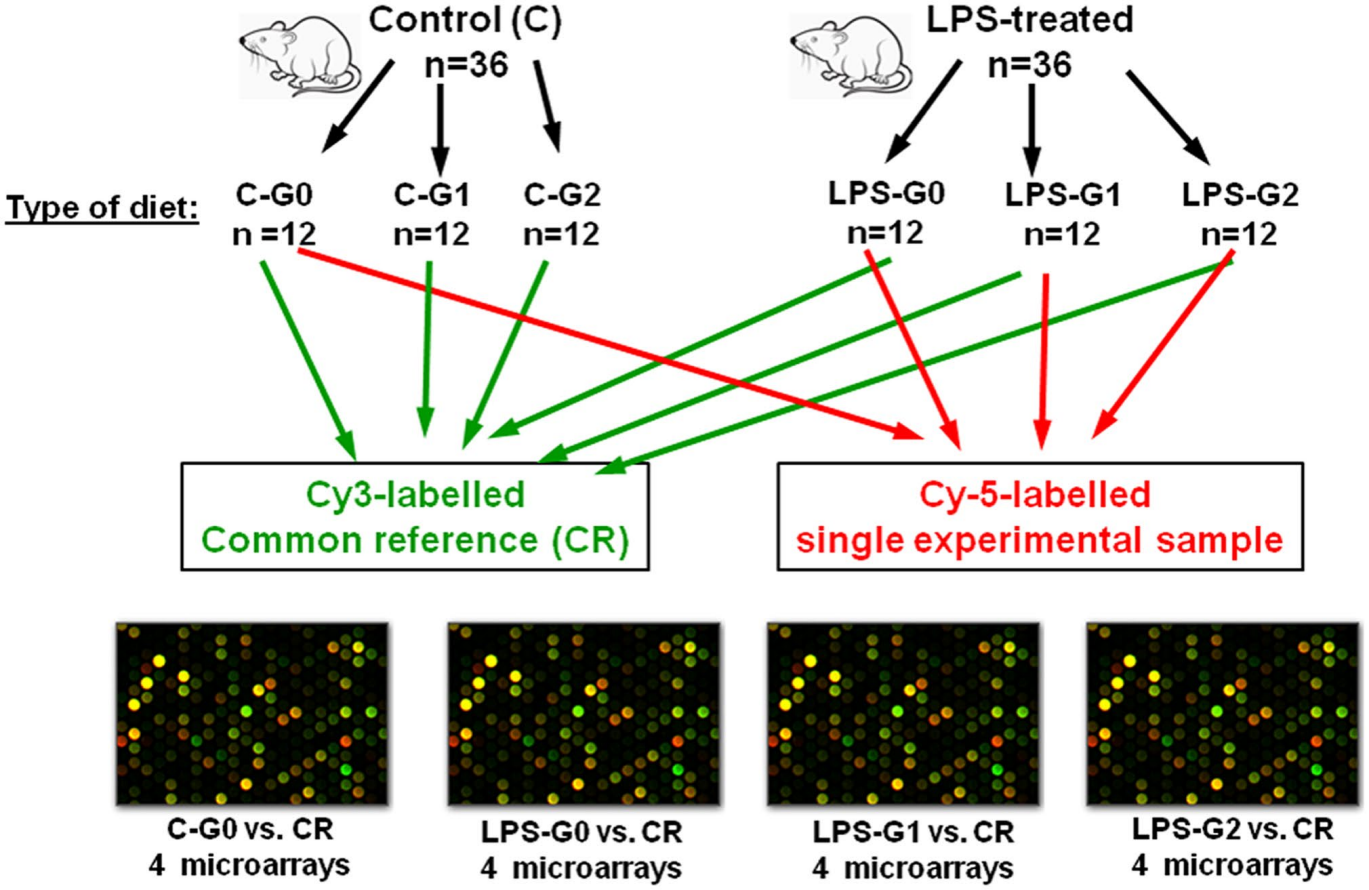
Scheme of experimental design used in the microarray analysis described in the study

The analysis of gene expression profile was performed using SurePrint G3 Rat Gene Expression Microarray, 8 × 60 K (Agilent Technologies, USA). The Low Input Quick Amp Labeling Kit (Agilent, USA) was used to amplify and label target RNA to generate complementary RNA (cRNA) for oligo microarrays used in gene expression profiling. Two-color microarrays were used. The experiment was performed using a common reference design, in which the common reference comprised a pool of equal amounts of RNA from each experimental group of animals (three samples per group). RNA samples included in the common reference pool were not used as the experimental samples in the microarray experiment. The cRNA of common reference was labeled with Cy3 and the cRNA isolated from blood of single rats from each experimental group: C-G0, LPS-G0, LPS-G1, and LPS-G2 were labeled with Cy5. For the purpose of the microarray analysis presented in this article, 16 two-color microarrays were performed: 4 microarrays for each experimental group (C-G0, LPS-G0, LPS-G1, LPS-G2) (Fig. 1).

On each microarray, 300 ng of Cy3-labeled cRNA of common reference sample and 300 ng of Cy5-labeled cRNA of control (C-G0) or LPS-treated experimental conditions (LPS-G0, LPS-G1, and LPS-G2) were hybridized. Microarray hybridization was performed with the Gene Expression Hybridization Kit (Agilent Technologies, USA), according to the manufacturer’s protocols. RNA Spike In Kit (Agilent Technologies, USA) was used as an internal control. Acquisition and analysis of hybridization intensities were performed using an Agilent DNA microarray scanner.

#### Signal detection and statistical analysis

After microarray scanning, data were extracted and background subtracted using the standard procedures contained in the Agilent Feature Extraction (FE) Software version 10.7.3.1. FE performs a Lowess normalization. To reveal the differential gene expression, statistical analysis was performed using the Gene Spring 12 software (Agilent, USA). The samples underwent quality control, and in the next step probe sets were filtered by flags to remove poor quality probes (absent flags). The statistical significance of the differences was evaluated using a one-way ANOVA and Tukey’s HSD post-hoc test (*p* < 0.05). A multiple testing correction was performed using Benjamini and Hochberg False Discovery Rate (FDR) < 5%. Microarray data were deposited at the Gene Expression Omnibus data repository under the number GSE102071 (https://www.ncbi.nlm.nih.gov/geo/query/acc.cgi?acc=GSE102071).

Further analysis was performed using the list of genes that showed at least twofold change in expression (FC ≥ 2). To identify signaling pathways and gene function, the microarray data were analyzed using Pathway Studio 6.0 (Ariadne Genomics). This is a database consisting of millions of individually modeled relationships between proteins, genes, complexes, cells, tissues, drugs, and diseases [15].

#### Real-time RT-PCR

To verify the microarray results, the expression of chosen genes from each compared experimental group was checked by real-time RT-PCR. Sequences of the chosen 17 genes were obtained from Ensembl or NCBI database. Primers were designed using the Primer-Blast software (NCBI database) and verified using Oligo Calc: Oligonucleotide Properties Calculator (free software available online, provided by Northwestern University) to exclude sequences showing self-complementarity. The secondary structures of the amplicon were examined by the mfold Web Server (free online access). *GAPDH* was used as the reference gene. The sequences of all primers used are listed in Table 1.

**Table 1:** Primer sequences for quantitative real-time PCR verification of microarray results

	Forward primer (5′–3′)	Reverse primer (5′–3′)
Target gene (LPS-G0 vs. C-G0)		
*Prodh*	GCTGCAGAGGATGGATGTG	GTAGCACTGGAACGTGTTGA
*Camsap2*	AGCCAGTCCAGTCCTGATAA	GAGTCCTCGTCCATGGTTTC
*Hmcn2*	GCGGATTCAGGGTCTACAAG	CTCCAGCTGTGTTGCTAACT
*Serp2*	GAGGGGAAACGTAGCCAAAAC	TTATGTGGCCAACCTGCAAAC
*Gzmc*	GCTTCCTGGTGAGAGACAAC	AGTCCTCTTGGCACTTCTCA
Target gene (LPS-G1 vs. LPS-G0)		
*Slc27a2*	CTACAACATCCGTGCCAAGT	GTGTCCACGCCATTAGTGTT
*Tpx2*	GAAGAGCAGAAGCAGCAGAA	GCCTCCATCTCAGCCATTTT
*Flt1*	TGGCTCCACGACCTTAGAC	GGCACCTATAGACACCCTCAT
*Serpini2*	CAGCAGCAGATCATGCAAAC	CCAAGACCAGACGAGTCAAG
*Clcn3*	CCTAGATGGAGCAGGTGCTAT	GATCCGTCTGTGCCTTTCTC
*Fat2*	GGAATTTCAGGGTGGCATGA	GAACGAGTATCGGCCATGAG
Target gene (LPS-G2 vs. LPS-G0)		
*Kcnk2*	ATGAACCCACGGGCGAAAA	AGTTCTGAGCAGCAGACTTG
*Mcm2*	AGAGACTACCGTCCCATTCC	TCAGCTCCTCCACATCTTCA
*Mtss1l*	GACAAGGACCATGCGAAAGA	CAGGTCTCCTTTCCCTTTGC
*Gas7*	ATGGCAACGGCACTTCAG	ACATTCCCAGGTGGTCTCAT
*Usp6nl*	TCCGGATCTGGGACATCTAC	GCCCGCTTTAACTCTGTCAT
*Efr3b*	AGATGTGGGTCGCCATAGAT	GCTTTCCACGAAGAGGTTGA
Reference gene		
*GAPDH* (*glyceraldehyde 3-phosphate dehydrogenase*)	GCTGGGGCTCACCTGAAGG	GGATGACCTTGCCCACAGCC

Total RNA was reverse transcribed to first-strand complementary DNA (cDNA) using the High-Capacity cDNA Reverse Transcription (Applied Biosystems, USA). All analyses were performed on the individual samples of total RNA using SYBR Select Master Mix (Applied Biosystems, USA) on Stratagene Mx3005P Quantitative PCR instrument for RT-PCR, following the manufacturer’s protocol. Annealing temperature depended on the pair of primers used to amplify specific genes. Real-time RT-PCR was performed on RNA samples from rats that were not used in the microarray experiment, but belonged to the analyzed groups (*n* = 5 per experimental group), and the reactions were performed in three repetitions per sample. The relative expression of the target genes was calculated using the 2^−ΔΔ*C*T^ method [16]. Results are presented as fold change over control (ratio: B/A; where B represents experimental conditions, A represents control conditions, i.e.: in LPS-G0 vs. C-G0, A represents control; in LPS-G1 vs. LP-G0 and in LPS-G2 vs. LPS-G0, A represents LPS-G0) and expressed as means ± SD. Data were analyzed by GenEx v.6 (bioMCC—bioscience Marketing Communication Consulting) and GraphPad Prism™ version 5.00 (GraphPad Software, Inc., USA) softwares.

## Results

In terms of the total feed intake and body weight gain, no statistically significant differences were noted among particular control (C) and *enteritis* (LPS) groups of animals, and these results have been published in our previous paper [16]. However, histopathological examination of the intestinal wall showed the clear signs of inflammation in the mucosa and submucosa layers, as was demonstrated in our previously published report [4].

In the present study, to determine the effect of two oat beta-glucan preparations of different molecular weight (G1: high-molecular-weight beta-glucan, 2 180 000 g/mol; G2: low-molecular-weight beta-glucan, 70 000 g/mol) on the gene expression profile in the peripheral blood of rats intravenously injected with LPS, we performed microarray analysis. The analysis revealed: 138 differentially expressed transcripts between the LPS-G0 and C-G0 groups, of which 45 genes were up-regulated and 93 were down-regulated in the LPS-G0 group. When the expression of genes was compared between the LPS-G1 and LPS-G0 groups, 533 transcripts showed differential expression, with 275 genes up-regulated and 258 genes down-regulated in the LPS-G1 group. The microarray analysis comparing the expression of genes between the LPS-G2 and LPS-G0 groups revealed 97 differentially expressed transcripts, of which 49 were up-regulated and 48 were down-regulated in the LPS-G2 group. The results presented include genes that were up-, or down-regulated more than twofold (fold change—FC ≥ 2). The results obtained are summarized in Table 2. Detailed information about all differentially expressed genes are presented in Supplementary Tables 1–3.

**Table 2:** Number of differentially expressed genes [*p* < 0.05 and fold change (FC) > 2] in peripheral blood of rats from different experimental groups

Groups	LPS-G0	LPS-G1	LPS-G2
C-G0	138	–	–
LPS-G0	–	533	97

Gene expression was detected by microarray analyses comparing the following sets of experimental conditions: LPS-G0 vs. C-G0 (LPS-challenged rats vs. control rat, both fed control diet); LPS-G1 vs. LPS-G0 (LPS-challenged rats fed diet supplemented with high-molecular-weight beta-glucan vs. LPS-challenged rats fed control diet); LPS-G2 vs. LPS-G0 (LPS-challenged rats fed diet supplemented with low-molecular-weight beta-glucan vs. LPS-challenged rats fed control diet)

### Validation of microarray data

**Fig. 2:**
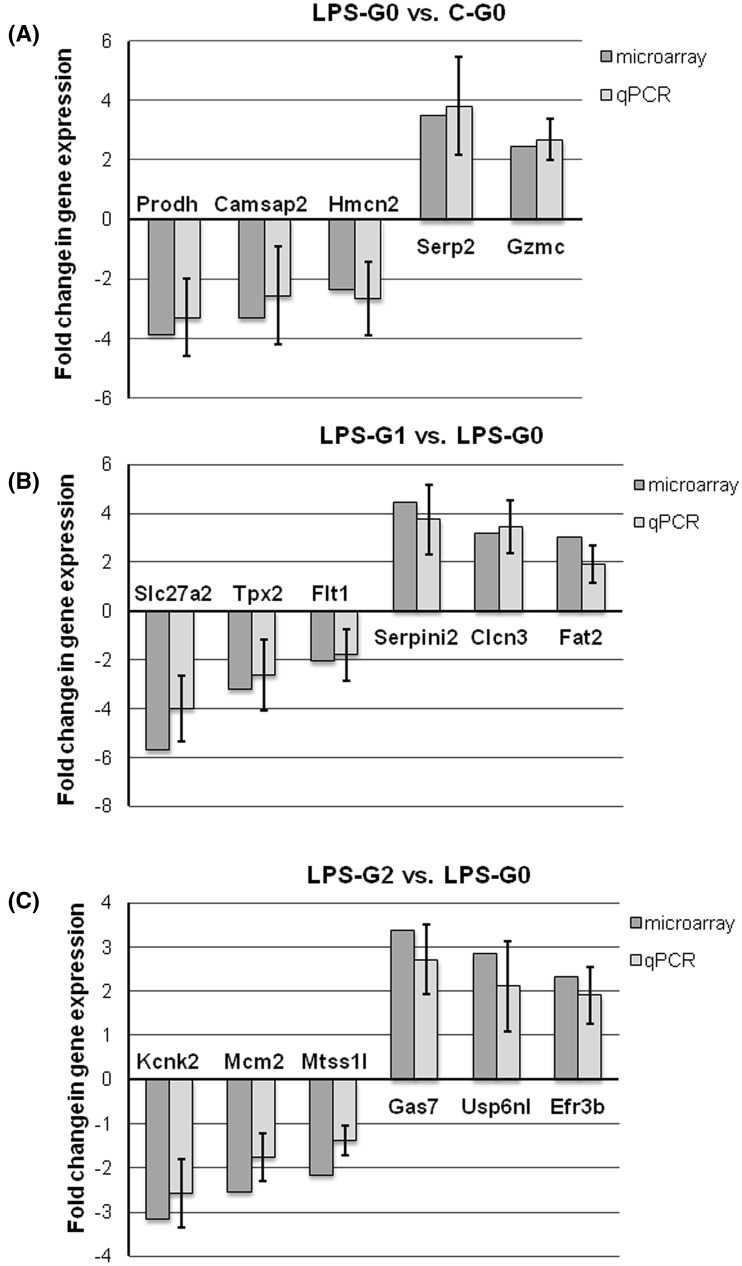
Expression of chosen genes in peripheral blood of rats: **a** with *enteritis* induced by intravenous LPS injection vs. healthy control rats (LPS-G0 vs. C-G0); **b** with *enteritis* induced by intravenous LPS injection, fed diet supplemented with G1 beta-glucan vs. LPS-challenged rats fed non-supplemented diet (LPS-G1 vs. LPS-G0); **c** with *enteritis* induced by intravenous LPS injection, fed diet supplemented with G2 beta-glucan vs. LPS-challenged rats fed non-supplemented diet (LPS-G2 vs. LPS-G0). Gene expression was analyzed using microarray and real-time PCR. Four microarrays were done per experimental condition and the results of fold change (FC) are statistically significant with the level of significance *p* < 0.05. Real-time RT-PCR results are presented as mean fold change ± SD of five samples per group in three repetitions per sample

To validate the microarray data, we selected 17 genes that showed differential expression between the examined experimental conditions, and amplified these genes using real-time RT-PCR. Genes selected for validation showed different levels of changes in expression (both: increased and decreased expressions) in each of the microarray performed, so that the list included highly regulated genes (showing over threefold change in expression), and those that were ≥ twofold up-, or down-regulated (representing FC close to the cut-off value = 2). We chose to analyze the expression of five genes when comparing the LPS-G0 vs. C-G0 conditions (three down-regulated and two up-regulated based on the microarray results); whereas, in the comparison between the LPS-G1 vs. LPS-G0 and LPS-G2 vs. LPS-G0, six genes were analyzed each time (three down-regulated and three up-regulated, based on the microarray results) (Table 1). The results obtained confirmed the direction of changes in gene expression in all of the analyzed transcripts (Fig. 2).

### Genes commonly regulated in peripheral blood of rats from both dietary groups supplemented with beta-glucans (LPS-G1 and LPS-G2)

A detailed analysis revealed that some of the differentially expressed genes were commonly detected in two or three compared conditions (Tables 3, 4). Within the group of genes with changed expression in the analysis comparing LPS-G0 vs. C-G0 and LPS-G1 vs. LPS-G0 or LPS-G2 vs. LPS-G0, the transcripts that were up-regulated in LPS-challenged rats fed control diet (LPS-G0) showed an opposite regulation (down-regulation) in the LPS-G1 (11 genes) and LPS-G2 (3 genes) conditions; whereas the transcripts down-regulated upon the LPS treatment (LPS-G0) showed increased expression in samples from LPS-G1 (23 genes) and LPS-G2 (5 genes) conditions (Table 3). This opposite tendency in expression indicates that these genes may be directly regulated in the course of the inflammatory process, and the presence of dietary beta-glucans may reverse the direction of their expression.

**Table 3:** List of genes reversely regulated in the group of rats with LPS-induced *enteritis* and fed semi-synthetic diet without beta-glucans (LPS-G0) in comparison to groups with LPS-induced *enteritis* fed diet supplemented with beta-glucans (LPS-G1 and LPS-G2)

Gene symbol		LPS-G0 vs. C-G0		LPS-G1 vs. LPS-G0		LPS-G2 vs. LPS-G0	
		Fold change	Direction of changes in expression	Fold change	Direction of changes in expression	Fold change	Direction of change in expression
1	*Dmtn*	2.047	Up	3.041	Down	Unchanged	
2	*Gpr52*	2.156	Up	2.304	Down	Unchanged	
3	*Gtsf1*	2.233	Up	2.171	Down	Unchanged	
4	*Krtap3-1*	2.183	Up	2.304	Down	Unchanged	
5	*Olr1406*	3.577	Up	3.281	Down	Unchanged	
6	*Pced1b*	2.187	Up	2.348	Down	Unchanged	
7	*RGD1563451*	3.131	Up	2.526	Down	2.688	Down
8	*RGD1564836*	2.113	Up	2.373	Down	2.268	Down
9	*RGD1566248*	2.230	Up	Unchanged		2.271	Down
10	*Serp2*	3.388	Up	12.594	Down	Unchanged	
11	*Vom1r8*	2.127	Up	2.305	Down	Unchanged	
12	*Brpf1*	2.357	Down	2.278	Up	Unchanged	
13	*Cars*	2.225	Down	2.097	Up	Unchanged	
14	*Ccdc38*	2.342	Down	4.115	Up	Unchanged	
15	*Dppa3l1*	2.018	Down	2.096	Up	Unchanged	
16	*Epm2aip1*	2.230	Down	2.040	Up	Unchanged	
17	*Fam172a*	2.721	Down	3.233	Up	Unchanged	
18	*Lysmd4*	2.168	Down	Unchanged		2.222	Up
19	*Mbp*	2.325	Down	2.647	Up	Unchanged	
20	*Mpp5*	2.067	Down	2.020	Up	Unchanged	
21	*Phf20l1*	2.140	Down	2.563	Up	Unchanged	
22	*Pip5k1a*	4.299	Down	2.047	Up	Unchanged	
23	*Pptc7*	2.179	Down	2.014	Up	Unchanged	
24	*Prodh*	3.859	Down	2.777	Up	8.065	Up
25	*Rab11fip1*	2.152	Down	2.463	Up	2.119	Up
26	*RGD1559908*	2.357	Down	2.777	Up	Unchanged	
27	*Scai*	2.367	Down	2.033	Up	Unchanged	
28	*Sft2d3*	2.845	Down	2.470	Up	Unchanged	
29	*Sik1*	3.268	Down	2.838	Up	Unchanged	
30	*Ssbp2*	2.088	Down	2.380	Up	Unchanged	
31	*Syap1*	2.316	Down	2.289	Up	Unchanged	
32	*Twistnb*	2.122	Down	2.296	Up	Unchanged	
33	*Usp6nl*	2.605	Down	Unchanged		2.835	Up
34	*Uvssa*	2.079	Down	Unchanged		2.273	Up

The list presents genes whose expression was significantly changed

**Table 4:** List of genes commonly regulated by beta-glucans supplementation in comparison to diet non-supplemented with beta-glucans (LPS-G0)

Gene symbol		LPS-G1 vs. LPS-G0		LPS-G2 vs. LPS-G0	
		Fold change	Direction of changes in expression	Fold change	Direction of change in expression
1	*Atg10*	2.319	Up	2.023	Up
2	*Ddx25*	2.750	Up	2.517	Up
3	*Efr3b*	2.155	Up	2.328	Up
4	*Fam83c*	2.529	Up	2.187	Up
5	*Fut4*	2.700	Up	2.161	Up
6	*Guf1*	2.546	Up	2.122	Up
7	*Mtus1*	2.964	Up	3.259	Up
8	*Olr1239*	2.373	Up	2.349	Up
9	*Rgsl1*	2.684	Up	2.426	Up
10	*She*	2.545	Up	2.538	Up
11	*Spetex-2F*	2.138	Up	2.353	Up
12	*Spg20*	3.397	Up	3.077	Up
13	*Zbtb48*	2.654	Up	2.150	Up
14	*Clybl*	2.602	Down	2.357	Down
15	*Cst12*	2.380	Down	2.730	Down
16	*Itga7*	2.277	Down	2.409	Down
17	*Olr1486*	2.517	Down	2.905	Down
18	*Spink14*	2.057	Down	2.215	Down
19	*Ube2c*	5.648	Down	3.550	Down

The list presents genes whose expression was significantly changed (*p* < 0.05 and fold change > 2)

In addition, there was also a group of 19 genes with common direction of changes in expression (up- or down-regulation) in the analyses comparing LPS-G1 vs. LPS-G0 as well as LPS-G2 vs. LPS-G0 (Table 4), indicating that these genes are specifically regulated by high- and low-molecular-weight beta-glucans.

### Signaling pathways involving the proteins encoded by differentially expressed genes

To identify the signaling pathways involving the proteins encoded by differentially expressed genes, we analyzed the microarray data using Pathway Studio 6.0 (Ariadne Genomics). This analysis revealed five cellular signaling pathways significantly regulated (*p* < 0.05) by G1 beta-glucan (Table 5), and four cellular signaling pathways significantly regulated (*p* < 0.05) by G2 beta-glucan (Table 6). Interestingly, among the statistically significant signaling pathways involving protein products of genes differentially expressed after G1 beta-glucan treatment, two were connected with the immune response of the body, namely: the NLR (nucleotide-binding domain, leucine-rich) proteins pathway, and the Toll-like receptor signaling pathway (Table 5). On the other hand, G2 beta-glucan induced signaling pathways connected with cell cycle regulation (cell cycle, G1 to S cell cycle control) and only one pathway directly connected with the immune response (prostaglandin synthesis and regulation) (Table 6). The results of this analysis suggest that high- and low-molecular-weight beta-glucans may induce different molecular mechanisms in organisms exposed to inflammatory stimuli such as LPS.

**Table 5:** List of cellular signaling pathways significantly regulated by proteins encoded by genes differentially expressed in peripheral blood of rats intravenously injected with LPS and fed diet supplemented with G1 beta-glucans in comparison to LPS-treated group fed control diet (LPS-G1 vs. LPS-G0)

Pathway	*p* value	Number of genes
NLR_Proteins_WP1294_71833	0.012	2
p53_pathway_WP655_78065	0.018	4
Cholesterol_Biosynthesis_WP461_71765	0.042	2
Toll-like_receptor_signaling_pathway_WP1309_72183	0.046	5
Nucleotide_Metabolism_WP146_71804	0.052	2

**Table 6:** List of cellular signaling pathways significantly regulated by proteins encoded by genes differentially expressed in peripheral blood of rats intravenously injected with LPS and fed diet supplemented with G2 beta-glucans in comparison to LPS-treated group fed control diet (LPS-G2 vs. LPS-G0)

Pathway	*p* value	Number of genes
Prostaglandin_Synthesis_and_Regulation_WP303_71789	0.024	1
G1_to_S_cell_cycle_control_WP348_71777	0.030	2
Non-homologous_end_joining_WP1277_69423	0.033	1
Cell_cycle_WP429_71805	0.048	2

## Discussion

In the present study, we used the animal model of rats challenged with intravenous injection of LPS to induce systemic inflammation and *enteritis* [4, 8]. LPS treatment has been shown to activate the innate immune response to bacterial infections [17]. In the bloodstream, LPS are detected by transmembrane Toll-like receptors (TLR4) located within the cell membrane of many types of immune cells, e.g., monocytes, neutrophils, and B cells [18]. This results in a stimulation of the inflammatory response manifested by the production and release of various inflammatory molecules, such as: interleukins (IL), tumor necrosis factor alpha (TNF-α), leukotrienes, nitric oxide (NO), or reactive oxygen species (ROS) [19]. Our study focused on the changes in gene expression found in the peripheral blood of LPS-challenged rats receiving different molecular weight oat beta-glucans in their diet in comparison to animals fed a non-supplemented diet. Microarray analyses compared gene expression in three different combinations: samples from LPS-challenged and non-challenged rats fed control diet (LPS-G0 vs. C-G0), samples from LPS-challenged rats fed diet enriched with high-molecular-weight beta-glucan vs. control diet (LPS-G1 vs. LPS-G0), and samples from LPS-challenged rats fed diet supplemented with low-molecular-weight beta-glucan vs. control diet (LPS-G2 vs. LPS-G0). Results obtained revealed a substantial number of differentially expressed mRNAs in each of the pairs compared.

### Genes related to LPS-induced inflammation

Among the genes up-regulated in the peripheral blood of LPS-challenged rats, we found mRNA encoding four granzymes: A, B, C, and K (*Gzma, Gzmb, Gzmc, and Gzmk*, respectively) and a granzyme-like protein 1 (LOC102553861) (Supplementary Table 1). Granzymes belong to the family of serine proteases expressed by cytotoxic T lymphocytes (Tc) and natural killer (NK) cells. These proteases serve to protect an organism against viral infections and cellular transformation [20]. They are known to induce apoptotic cell death via the granule exocytosis pathway [21]. However, an increasing body of evidence indicates that granzymes may also have other functions in inflammation, i.e., granzymes A, B, and K may be involved in the production, release and/or processing of proinflammatory cytokines [22]. Studies on mice have shown that granzyme A has a direct role in the inflammatory response to LPS [23], whereas the extracellular form of this protein activates primary human monocytes to release TNF-α, IL-1β, IL-6, and IL-8 [24]. Similarly, granzyme B has been shown to enhance LPS-induced TNF-α release from monocytes in vitro; however, it was not able to exert this effect when administered alone [25]. The recombinant murine granzyme K induced the release of IL-1β from peritoneal mouse macrophages, and this effect was observed after priming the cells with LPS, suggesting an additive or synergistic effect of granzymes and bacterial compounds [26]. The role of granzyme C is less clear, although its expression was detected in activated CD4 + and CD8 + T lymphocytes, as well as in NK cells stimulated with IL-15 [27]. The transcriptomic analysis performed in our study also confirmed that intravenous administration of LPS to rats caused a systemic inflammatory response. Interestingly, a comparison of the gene expression profile of the LPS-G1 vs. LPS-G0 groups revealed a 2.35-fold decrease in the expression of gene encoding the granzyme C-like protein (RGD1560650), pointing to potential antiinflammatory activity of G1 beta-glucan in the LPS-challenged rats supplemented with this glucose polymer (Supplementary Table 2). Even more pronounced decrease (12.594-fold) in expression was noted in the case of *Serp2* gene in rats from LPS-G1 group. The same gene was 3.388 up-regulated in LPS-challenged rats fed the control diet (LPS-G0) (Table 3, Supplementary Tables 1 and 2). *Serp2* encodes stress-associated endoplasmic reticulum protein family member 2, belonging to the family of serine protease inhibitors, termed serpins, which are key regulators of numerous biological pathways involved in initiation of inflammation, coagulation, angiogenesis, apoptosis, extracellular matrix composition, and complement activation responses [28]. The exact function of Serp2 expressed by mammalian species has not been fully elucidated so far; however, Messud-Petit and co-workers [29] showed that Serp2 specifically binds to IL-1β-converting enzyme (ICE), and can prevent the onset of inflammation by reducing the levels of the active proinflammatory cytokine IL-1β. Therefore, the weakening of the immune response is linked to *Serp2* gene function. To our knowledge, this is the first report showing a strong effect of beta-glucans on *Serp2* gene expression, strongly supporting the immunomodulatory activities of these polysaccharides.

### Genes related to immunomodulatory functions of beta-glucans

Among the 533 genes regulated in the blood of rats from the LPS-G1 group in comparison with the LPS-G0 group, several other transcripts were directly connected with the host immune response. Among the 275 up-regulated genes listed in Supplementary Table 2, special attention should be paid to those encoding: TNF receptor superfamily member 5 (*CD40*); immunoglobulin lambda-like polypeptide 1 (*Igll1*); immunoglobulin delta heavy chain secreted form (*IgD*) mRNA; Fc fragment of IgE, low affinity II receptor for (CD23) (*Fcer2*); TNF receptor-associated factor 3 (*Traf3*) and NLR family, pyrin domain-containing 1B protein (*Nlrp1b*). Products of these genes are involved in the signaling pathways induced in response to inflammatory processes, such as the TLR signaling pathway and the NLR signaling pathway (Table 5). Currently, increasing number of data demonstrate that beta-glucans are able to regulate both innate and adaptive immunity. Macrophages and dendritic cells are considered the main target cells of beta-glucans, although neutrophils, B cells, T cells, and natural killer cells are also known to be activated by these polysaccharides [30]. Beta-glucans, which cannot directly penetrate cell membrane due to their large molecular size [31], may act as pathogen-associated molecular patterns (PAMPs) that are recognized by cell surface receptors called pattern recognition receptors (PRRs) [32]. Among PRRs that are best known to bind beta-glucans are dectin-1 and the Toll-like receptors (TLRs). Binding of beta-glucans to dectin-1 and TLRs induces intracellular signaling pathways which play an important role in the innate immune response [33, 34]. Beta-glucan-activated signaling cascades eventually lead to the release of cytokines, including IL-12, IL-6, TNF-α, and IL-10 [35].

Our study revealed that *CD40* was among the genes connected with TLR-induced signaling pathways, which were up-regulated in LPS-G1 group when compared to LPS-G0 group. CD40 protein is a member of the TNF receptor (TNFR) superfamily recognizing CD40L molecule (Supplementary Table 2). CD40L is produced by both Th1 and Th2 helper T cells, as well as by mast cells, basophils, and eosinophils [36]. Signals induced by CD40L expressed in Th1 and Th2 cells are known to be required for macrophage and B cell activation, respectively [37]. The production of IgE by B cells is induced by CD40-stimulated ERK kinase activation transduced via serine/threonine protein kinase Tpl2 [37]. Interestingly, our study also demonstrated increased expression of other genes connected with TLR signaling and B cell activity. Genes encoding TNF receptor-associated factor 3 (Traf3), and immunoglobulins: IgD, Igll1, and the Fc fragment of IgE (Fcer2) were up-regulated in the LPS-G1 group when compared with the LPS-challenged group (LPS-G0). These results may indicate that dietary supplementation with high-molecular-weight oat beta-glucan in animals with an LPS-induced inflammatory state resulted in a modulation of the immune response of the organism, at least partially mediated by molecules involved in the TLR signaling pathway and B lymphocytes activation. In fact, our previous report on the effect of low- and high-molecular-weight oat beta-glucans on the inflammatory and oxidative stress status in the colons of rats with LPS-induced *enteritis* also demonstrated that G1 beta-glucan caused a significant increase in B cells within the population of intraepithelial lymphocytes [4]. At the same time, we observed a significant decrease in the levels of proinflammatory cytokines in the colon tissue lysates from rats with *enteritis* fed the G1 beta-glucan-enriched diet [4].

In addition to the changes in the expression of genes connected with the TLR signaling pathway, we also noted up-regulated levels of *Nlrp1b* mRNA (NLR family, pyrin domain-containing 1B), which is involved in the NLR signaling pathway (Supplementary Table 2). In animals, the NLR protein family is a class of innate immune molecules comprising PRRs localized in the cellular cytoplasm to recognize microbial products. Among these proteins, NOD1 and NOD2 are known to detect bacterial cell wall components [38]. The activation of NLR proteins results in inflammatory responses mediated by NFκB, MAPK or caspase-1 activation, accompanied by subsequent secretion of proinflammatory cytokines [38]. Other proteins belonging to the NLR family are involved in the formation of inflammasomes in cells. Inflammasomes are macromolecular scaffolds that are composed of an NLR, the adaptor protein ASC, and caspase-1, which form in the cytosol following NLR activation in response to specific microbe- and/or damage-associated molecular patterns [39]. As the sensor component of the inflammasomes, NLRP1 plays a crucial role in innate immunity and inflammation. In response to pathogens and other damage-associated signals, NLP1 initiates the formation of the inflammasome polymeric complex [40]. Furthermore, NLRP1 has been shown to be important in attenuating gastro-intestinal inflammation and tumorigenesis [39]. It was, therefore, interesting to observe that supplementing the diet of LPS-challenged rats with G1 beta-glucan elevated the expression of *Nlrp1*, providing another argument to the hypothesis that beta-glucans have protective properties against inflammation. It should be noted, however, that the increase in *Nlrp1* expression was detected only in the case of the G1 beta-glucan, but not in the case of the low-molecular-weight G2 beta-glucan.

A comparison of the peripheral blood transcriptomic profile between the LPS-G2 and LPS-G0 groups revealed the smallest number of differentially expressed genes (Table 2). These findings are in line with the other results obtained in the course of our studies on the role of dietary supplementation with beta-glucans in rats with LPS-induced *enteritis*, which all showed that low-molecular-weight beta-glucan (G2) was the less potent modulator of the immune response [4, 8]. In general, large molecular weight beta-glucans are regarded more biologically active, whereas short beta-glucans with molecular weight below 5000–10,000 Da were shown to be inactive [41]. Furthermore, soluble beta-glucans appear to be stronger immunostimulators than insoluble ones [30]. Our observations confirm these findings, as the high-molecular-weight beta-(1 → 3)/(1 → 4)-linked oat d-glucan showed better antiinflammatory and antioxidative potential in colon and peripheral blood of rats challenged with LPS [4, 8]. This is at least partially connected with its physical property, i.e., viscosity and solubility, in the intestine, where the organism is first exposed to beta-glucans action.

Although, in our present study, a substantially lower number of genes connected with immune response were regulated under the influence of low-molecular-weight oat beta-glucan (G2), it is worth noting that *IL34* mRNA showed increased expression in the LPS-G2 group when compared to LPS-G0 group (Supplementary Table 3). Interleukin 34 (IL-34) promotes the proliferation, survival, and differentiation of monocytes and macrophages. It is also known to stimulate the release of proinflammatory chemokines, and thereby plays an important role in innate immunity and in inflammatory processes [42]. Foucher et al. [43] have shown that IL-34 induces the differentiation of monocytes into resident macrophages (MΨ). Their results have demonstrated that IL-34-stimulated macrophages have potent immunosuppressive properties. Upon LPS stimulation, those MΨ macrophages treated with IL-34 produced high levels of antiinflammatory IL-10 and low levels of proinflammatory IL-12 [43]. To our knowledge, our report is the first showing a possible relationship between beta-glucans and *IL-34* expression; however, Sarinho et al. [44] demonstrated that subcutaneous beta-(1 → 3)-d-glucan injections applied to children with mild-to-moderate persistent asthma resulted in the patients having increased serum IL-10 levels. Those authors suggested that beta-glucans may be used to modulate allergic sensitisation and may be beneficial in restoring Th2 cells function.

A comparison of the gene expression profile between the LPS-G2 and LPS-G0 groups also revealed a 2.7-fold down-regulation of the prostaglandin E receptor 3 (*PTGER3*) mRNA levels in the blood of the LPS-challenged rats supplemented with G2 beta-glucan (Supplementary Table 3). This receptor is involved in the prostaglandin E2 signaling pathway (Table 6). So far, the majority of data suggest that prostaglandin E receptor (EP3) mostly inhibits immune responses. In an OVA-induced asthma model, inflammation was more pronounced in EP3-deficient mice than in wild-type mice, and an EP3 agonist suppressed inflammation [45]. EP3 stimulation also resulted in the inhibition of skin inflammation in a contact hypersensitivity model [46]. Further studies are necessary to determine the possible relationship between the molecular activity of beta-glucans and decreased *PTGER3* expression.

### Genes connected with anticancer properties of beta-glucans

Among genes commonly regulated in both groups supplemented with beta-glucans (LPS-G1 and LPS-G2), two genes particularly have drawn our attention: *Mtus1* (microtubule associated tumor suppressor 1) which was significantly up-regulated, and *Ube2C* (ubiquitin-conjugating enzyme E2C) which was significantly down-regulated by both beta-glucans (Table 4). Data in the literature show that the expression of *Mtus1* is down-regulated in a wide range of cancers: colon tumors, breast cancer, ovarian cancer, head and neck squamous cell carcinoma, as well as prostate cancer cell lines [47–50]. Transfections of HUVEC cells with *Mtus1* siRNA resulted in significantly increased cell proliferation, showing the important antimitotic properties of this gene [50]. On the other hand, *Ube2c* high expression has been shown to be positively correlated with unfavorable prognosis in various cancer types of: breast, bladder, lung, and thyroid gland [51–55]. UBE2C is an integral component of the ubiquitin proteasome system required for the destruction of mitotic cyclins and securin, which are essential for spindle assembly checkpoint and mitotic exit [51]. Cells overexpressing *Ube2c* ignore the mitotic spindle checkpoint signals and lose genomic stability, which is a hallmark of cancer [55]. In our study, the expression of *Ube2c* was over fivefold down-regulated in LPS-G1 group and over threefold down-regulated in LPS-G2 group when compared with LPS-G0 group (Table 4). So far, there are no data showing a direct relationship between beta-glucans and the aforementioned genes; however, the anticancer properties of beta-glucans have been widely suggested. The effect of beta-(1 → 3),(1 → 4)-d-glucan of high molecular weight in the form of a highly purified water extract was tested in vitro on human skin melanoma HTB-140 cells [56]. The results showed a concentration-dependent increase in apoptosis induction manifested by augmented activation of caspase-3/-7 and an increased number of cells positively stained with annexin V-FITC. The same compound of low molecular weight was used to test antitumor capacity in the following cancer cells: Me45, A431, and normal HaCaT and murine macrophages [57]. The study showed that beta-(1 → 3),(1 → 4)-d-glucan significantly decreased the viability of cancer cells in a dose- and time-dependent manner, while the polysaccharide was non-toxic for normal cells. The effect of both high- and low-molecular-weight beta-(1 → 3),(1 → 4)-d-glucans was also studied on human lung adenocarcinoma cells, human multidrug-resistant small cell lung cancer cells, and normal human keratinocytes, showing an induction of oxidative stress and the cytotoxic properties of beta-glucan preparations in cancer cells but not normal keratinocytes [58]. Furthermore, research on beta-glucans isolated from other sources (yeast and fungi) also provides the evidence of the anticancer properties of these compounds. Beta-(1 → 3)-d-glucan from *Saccharomyces cerevisiae* has been shown to exert antitumor effects in S180 tumor-bearing mice without toxicity in normal mouse cells. Treatment with beta-(1 → 3)-d-glucan resulted in an increased rate of tumor inhibition in a dose-dependent manner, and significantly potentiated immune responses in mice [59]. In another study, Maitake D-Fraction extracted from *Grifola frondosa* (Maitake mushroom) decreased the size of lung, liver, and breast tumors in > 60% of patients when it was combined with chemotherapy in a two-arm controlled study compared with chemotherapy alone [60]. In a prospective clinical trial of the short-term immune effects of oral beta-glucan in patients with advanced breast cancer, 23 female patients with advanced breast cancer were compared with a control group of 16 healthy females [61]. Beta-(1 → 3),(1 → 6)-d-glucan was taken daily by oral administration and blood samples were collected on days 0 and 15 of the trial. The results showed that, despite a relatively low initial white cell count, oral beta-glucan stimulated the proliferation and activation of peripheral blood monocytes in the patients with advanced breast cancer. In the present study, the up-regulation of *Mtus1* and significant down-regulation of *Ube2c* in the peripheral blood of the LPS-challenged rats supplemented with beta-glucans may support the hypothesis concerning the potential anticancer effects of these polysaccharides.

### Autophagy-related genes and beta-glucans

The microarray analyses conducted in the present study also revealed that the administration of oat beta-glucans in the diet resulted in increased expression of *Atg10* transcript, which belongs to the family of autophagy-related genes (*ATGs*) (Table 4). In addition, a comparison between the LPS-G1 and LPS-G0 experimental groups showed a significant up-regulation of another member of the *ATG* family, *Atg13* (Supplementary Table 2). Autophagy is a homeostatic process of lysosomal degradation observed in all eukaryotic organisms. It is based on the formation of double-membrane vesicles, termed autophagosomes, which engulf long-lived cytoplasmic proteins, damaged organelles and even invasive pathogens, and transport these cargos to the lysosomes in which the cargo is degraded by the hydrolytic enzymes [62]. Autophagy is constitutively active in cells at a basal level, providing a quality control mechanism for proteins and organelles to protect cells from the adverse consequences of aggregated or misfolded proteins, as well as damaged organelles, such as mitochondria, which could induce cell death or degenerative diseases. On the other hand, autophagy is regarded as an essential prosurvival catabolic pathway induced by a wide variety of stresses, including nutrient deprivation, hypoxia, oxidative stress, growth factor withdrawal, and intracellular infections. The process is strictly regulated by several signaling pathways, and remains under the control of over 30 *ATGs* and their protein products. Recently, an increasing number of reports have described the role of autophagy in adaptive immunity [63], as well as in the endogenous pathway of antigen MHC II presentation [64]. Both differentially expressed autophagic genes, *Atg10* and *Atg13*, encode proteins involved in the process of autophagy induction in cells. Currently, some reports describe a possible relationship between beta-glucans and autophagic activity in cells. Studies have shown that the recognition of beta-glucan by dectin-1 could trigger the formation of phagosomes that specifically recruit LC3, which is a protein associated with the membranes of autophagosomes, and, thus, regarded as a reliable marker of autophagy induction in cells [65]. Dectin-1 signaling was entirely sufficient to trigger LC3-associated phagocytosis of fungal beta-glucan particles [66]. Although there are no data describing the possible regulation of *ATG* expression by beta-glucans, our findings suggest that these polysaccharides may play a role in autophagy induction, and the connection between this process and immunomodulation should be further explored.

### Ion channel-related genes in the context of inflammation and beta-glucans supplementation

Finally, among the genes that were differentially expressed in the peripheral blood of the rats supplemented with beta-glucans, quite a substantial number of transcripts represented proteins involved in ion channel formation or regulation in cell membranes, e.g., *Kcng2, Kcnc4, Scn7a, Nkain4, Camk1g* (in LPS-G1 vs. LPS-G0 analysis); *Kcnmb2, Kcnj12* (in G2-LPS vs. LPS analysis) (for a gene description see Supplementary Tables 2 and 3). These genes encode proteins of potassium, sodium, and calcium voltage channels, and interestingly, they were all down-regulated in the beta-glucan-treated groups (Supplementary Tables 2 and 3). Some reports describe a relationship between the activity of ion-voltage channels and LPS. Gerth et al. [67] exposed macrophages to LPS to study the early cellular events associated with LPS-mediated macrophage activation. They used the whole-cell patch clamp technique to analyze ion channels and measure the membrane currents in relation to the translocation of the NFκB transcription factor to the nucleus. Exogenous LPS was shown to increase the voltage-dependent outward current, whereas the voltage-dependent inward current was unaffected [67]. In parallel, the authors found that exogenous LPS led to the nuclear localization of the p65 subunit of NFκB, proving the activation of this transcription factor, which is a major process in macrophage activation, as NFκB induces the expression of a number of genes encoding many inflammatory mediators [67]. Thus, it is possible that the down-regulation of genes encoding potassium and sodium channel proteins in the LPS-G1 and LPS-G2 experimental groups may also be connected with the antiinflammatory functions of beta-glucans and the attenuation of the inflammatory state induced by LPS injection. Furthermore, Schmid-Antomarchi et al. [68] demonstrated that the level of activity of NADPH oxidase is dependent on the plasmic membrane potential, and suggested a link between the generation of reactive oxygen intermediates and the opening of the K(Ca) channels. Our previous studies showed that the effect of beta-glucans was connected with their antioxidative activities, as rats with LPS-induced *enteritis* that were supplemented with beta-glucans had an increased concentration of antioxidative potential markers (TAS, SOD, GR, and GPx activity; TBARS concentration) in the blood, spleen, colon tissue, stomach, and liver [4, 8–10]. Taken all together, these findings may suggest that the decreased expression of genes connected with ion-voltage channel activity is linked with the antiinflammatory and antioxidative activity of beta-glucans observed in our in vivo experimental model.

## Summary

In summary, the microarray analysis performed in the present study describe many novel potential targets of the molecular actions of high- and low-molecular-weight oat beta-glucans administered via dietary supplementation. The majority of genes described are directly or indirectly linked with the immunomodulatory functions of beta-glucans. The direction of changes in gene expression in the peripheral blood indicates that beta-glucans exerted a protective effect in rats with an induced inflammatory state caused by LPS challenge. The results of our previous studies demonstrated that this protective effect of beta-glucans was less pronounced in animals under physiological conditions. Thus, it is possible that the efficacy of supplementation with beta-glucans may be higher when applied in the course of ongoing inflammation, rather than during prophylactic use. Further studies are needed to explore in details the role of those genes that were shown to be differentially expressed under the influence of beta-glucans.

## Electronic supplementary material

Below is the link to the electronic supplementary material.

Supplementary material 1 (DOCX 35 KB)

Supplementary material 2 (DOCX 77 KB)

Supplementary material 3 (DOCX 30 KB)
