# Supplementary material for: Oral administration of oat beta-glucan preparations of different molecular weight results in regulation of genes connected with immune response in peripheral blood of rats with LPS-induced enteritis
Source: Eur J Nutr. 2018 Oct 4;58(7):2859–73. doi: 10.1007/s00394-018-1838-3 (PMC6769091; doi:10.1007/s00394-018-1838-3)
Supplement: Supplementary file 1 — Supplementary material 1 (DOCX 35 KB) [file 394_2018_1838_MOESM1_ESM.docx]

**Supplementary Table 1.** List of genes differentially expressed in peripheral blood of LPS-treated rats treated with LPS i comparison to control animals (LPS-G0 vs. C). The list presents genes whose expression was significantly changed (p <0.05 and fold change (FC) >2). Genes described in discussion are bolded.

| GeneSymbol | p (Corr) | Regulation | FC (abs) | Description |
| --- | --- | --- | --- | --- |
| Fam111a | 0.029 | up | 5.372 | Rattus norvegicus family with sequence similarity 111, member A (Fam111a), mRNA [NM_001109163] |
| Mcm2 | 0.044 | up | 3.582 | Rattus norvegicus minichromosome maintenance complex component 2 (Mcm2), mRNA [NM_001107873] |
| Olr1406 | 0.043 | up | 3.577 | Rattus norvegicus olfactory receptor 1406 (Olr1406), mRNA [NM_001000786] |
| Serp2 | 0.038 | up | 3.495 | Rattus norvegicus stress-associated endoplasmic reticulum protein family member 2 (Serp2), mRNA [NM_001109104] |
| Atp2c2 | 0.043 | up | 3.388 | Rattus norvegicus ATPase, Ca++ transporting, type 2C, member 2 (Atp2c2), mRNA [NM_134462] |
| RGD1563451 | 0.035 | up | 3.313 | PREDICTED: Rattus norvegicus TD and POZ domain-containing protein 2-like (RGD1563451), mRNA [XM_003753628] |
| Fam111a | 0.023 | up | 3.094 | Rattus norvegicus family with sequence similarity 111, member A (Fam111a), mRNA [NM_001109163] |
| **LOC102553861** | 0.043 | up | 3.051 | PREDICTED: Rattus norvegicus granzyme-like protein 1-like (LOC102553861), mRNA [XM_006252056] |
| RGD1563562 | 0.048 | up | 2.730 | PREDICTED: Rattus norvegicus uncharacterized LOC295012 (RGD1563562), mRNA [XM_006232315] |
| Gabpb1 | 0.046 | up | 2.676 | Rattus norvegicus GA binding protein transcription factor, beta subunit 1 (Gabpb1), mRNA [NM_001039036] |
| Pkd1 | 0.038 | up | 2.473 | Rattus norvegicus polycystic kidney disease 1 homolog (human) (Pkd1), mRNA [NM_001257352] |
| **Gzmc** | **0.038** | **up** | **2.470** | **Rattus norvegicus granzyme C (Gzmc), mRNA [NM_134332]** |
| Fgf20 | 0.043 | up | 2.426 | Rattus norvegicus fibroblast growth factor 20 (Fgf20), mRNA [NM_023961] |
| Olr821 | 0.037 | up | 2.365 | Rattus norvegicus olfactory receptor 821 (Olr821), mRNA [NM_001000842] |
| LOC367545 | 0.046 | up | 2.362 | PREDICTED: Rattus norvegicus Y-linked testis-specific protein 1-like (LOC367545), partial mRNA [XM_003754692] |
| **Gzma** | **0.043** | **up** | **2.360** | **Rattus norvegicus granzyme A (Gzma), mRNA [NM_153468]** |
| LOC291276 | 0.043 | up | 2.353 | isopentenyl-diphosphate delta isomerase 2 (Idi2), mRNA [Source:RefSeq mRNA;Acc:NM_001192008] [ENSRNOT00000045035] |
| Sox13 | 0.046 | up | 2.330 | Rattus norvegicus SRY (sex determining region Y)-box 13 (Sox13), mRNA [NM_001105952] |
| Tex15 | 0.043 | up | 2.307 | Rattus norvegicus testis expressed 15 (Tex15), mRNA [NM_001106087] |
| LOC100363248 | 0.038 | up | 2.301 | SOGA family member 3 [Source:MGI Symbol;Acc:MGI:1914662] [ENSRNOT00000016712] |
| Eppk1 | 0.044 | up | 2.274 | PREDICTED: Rattus norvegicus epiplakin 1 (Eppk1), transcript variant X1, mRNA [XM_003754311] |
| Pipox | 0.046 | up | 2.273 | Rattus norvegicus pipecolic acid oxidase (Pipox), mRNA [NM_001012009] |
| St6galnac6 | 0.044 | up | 2.238 | Rattus norvegicus ST6 (alpha-N-acetyl-neuraminyl-2,3-beta-galactosyl-1,3)-N-acetylgalactosaminide alpha-2,6-sialyltransferase 6 (St6galnac6), mRNA [NM_001015036] |
| Gtsf1 | 0.046 | up | 2.233 | Rattus norvegicus gametocyte specific factor 1 (Gtsf1), mRNA [NM_001079707] |
| RGD1566248 | 0.038 | up | 2.230 | PREDICTED: Rattus norvegicus necdin-like (RGD1566248), mRNA [XM_006222985] |
| LOC685125 | 0.044 | up | 2.226 | PREDICTED: Rattus norvegicus disks large homolog 5-like (LOC685125), partial mRNA [XM_006255360] |
| Spink2 | 0.038 | up | 2.198 | Rattus norvegicus serine peptidase inhibitor, Kazal type 2 (acrosin-trypsin inhibitor) (Spink2), mRNA [NM_001008870] |
| Pced1b | 0.040 | up | 2.187 | Rattus norvegicus PC-esterase domain containing 1B (Pced1b), mRNA [NM_001039454] |
| Krtap3-1 | 0.043 | up | 2.183 | PREDICTED: Rattus norvegicus keratin associated protein 3-1 (Krtap3-1), mRNA [XM_001055567] |
| Gpr52 | 0.043 | up | 2.156 | Rattus norvegicus G protein-coupled receptor 52 (Gpr52), mRNA [NM_001289935] |
| RGD1560608 | 0.044 | up | 2.148 | Rattus norvegicus similar to novel protein (RGD1560608), mRNA [NM_001109280] |
| **Gzmk** | **0.041** | **up** | **2.139** | **Rattus norvegicus granzyme K (Gzmk), mRNA [NM_017119]** |
| Spns2 | 0.044 | up | 2.139 | Rattus norvegicus spinster homolog 2 (Spns2), mRNA [NM_001144991] |
| Vom1r8 | 0.043 | up | 2.125 | Rattus norvegicus vomeronasal 1 receptor 8 (Vom1r8), mRNA [NM_001008905] |
| Bag4 | 0.048 | up | 2.124 | Rattus norvegicus BCL2-associated athanogene 4 (Bag4), mRNA [NM_001025130] |
| RGD1564836 | 0.043 | up | 2.113 | Protein RGD1564836 [Source:UniProtKB/TrEMBL;Acc:D3ZAX1] [ENSRNOT00000038940] |
| Lekr1 | 0.040 | up | 2.110 | Rattus norvegicus similar to Restin, mRNA (cDNA clone MGC:187532 IMAGE:7455378), complete cds. [BC158627] |
| LOC102551633 | 0.044 | up | 2.086 | PREDICTED: Rattus norvegicus sperm motility kinase W-like (LOC102551633), mRNA [XM_006225975] |
| **Gzmb** | **0.048** | **up** | **2.072** | **Rattus norvegicus granzyme B (granzyme 2, cytotoxic T-lymphocyte-associated serine esterase 1) (Gzmb), mRNA [NM_138517]** |
| Tas1r1 | 0.044 | up | 2.064 | Rattus norvegicus taste receptor, type 1, member 1 (Tas1r1), mRNA [NM_053305] |
| RGD2301395 | 0.044 | up | 2.060 | Rattus norvegicus similar to killer cell lectin-like receptor subfamily B member 1A (RGD2301395), mRNA [NM_001135687] |
| Fam169a | 0.045 | up | 2.060 | PREDICTED: Rattus norvegicus family with sequence similarity 169, member A (Fam169a), transcript variant X3, mRNA [XM_006223988] |
| Dmtn | 0.048 | up | 2.047 | Rattus norvegicus dematin actin binding protein (Dmtn), mRNA [NM_001108385] |
| Ptger3 | 0.044 | up | 2.041 | Rattus norvegicus prostaglandin E receptor 3 (subtype EP3) (Ptger3), mRNA [NM_012704] |
| Cyp4a8 | 0.044 | up | 2.024 | Rattus norvegicus cytochrome P450, family 4, subfamily a, polypeptide 8 (Cyp4a8), mRNA [NM_031605] |
| Pip5k1a | 0.038 | down | 4.299 | Rattus norvegicus phosphatidylinositol-4-phosphate 5-kinase, type 1, alpha (Pip5k1a), mRNA [NM_001042621] |
| Prodh | 0.027 | down | 3.859 | Rattus norvegicus proline dehydrogenase (oxidase) 1 (Prodh), nuclear gene encoding mitochondrial protein, mRNA [NM_001135778] |
| Zfp395 | 0.035 | down | 3.631 | Rattus norvegicus zinc finger protein 395 (Zfp395), mRNA [NM_001107271] |
| Camsap2 | 0.029 | down | 3.289 | Rattus norvegicus calmodulin regulated spectrin-associated protein family, member 2 (Camsap2), mRNA [NM_001134503] |
| Sik1 | 0.046 | down | 3.268 | Rattus norvegicus salt-inducible kinase 1 (Sik1), mRNA [NM_021693] |
| LOC689520 | 0.040 | down | 3.233 | PREDICTED: Rattus norvegicus sperm motility kinase W-like (LOC689520), partial mRNA [XM_001071066] |
| RGD1565283 | 0.038 | down | 3.178 | Rattus norvegicus similar to novel protein (RGD1565283), mRNA [NM_001109074] |
| Tnks2 | 0.048 | down | 3.088 | Rattus norvegicus tankyrase, TRF1-interacting ankyrin-related ADP-ribose polymerase 2 (Tnks2), mRNA [NM_001107607] |
| Sfrp5 | 0.044 | down | 3.071 | Rattus norvegicus secreted frizzled-related protein 5 (Sfrp5), mRNA [NM_001107591] |
| Ahcyl1 | 0.040 | down | 3.044 | Rattus norvegicus adenosylhomocysteinase-like 1 (Ahcyl1), mRNA [NM_001108561] |
| Immp1l | 0.038 | down | 2.945 | PREDICTED: Rattus norvegicus IMP1 inner mitochondrial membrane peptidase-like (S. cerevisiae) (Immp1l), transcript variant X3, mRNA [XM_006224544] |
| Rnf145 | 0.038 | down | 2.909 | Rattus norvegicus ring finger protein 145 (Rnf145), mRNA [NM_001105778] |
| Pnkd | 0.027 | down | 2.862 | Rattus norvegicus paroxysmal nonkinesigenic dyskinesia (Pnkd), transcript variant 2, mRNA [NM_001134751] |
| Rnf186 | 0.043 | down | 2.857 | Rattus norvegicus ring finger protein 186 (Rnf186), mRNA [NM_001109592] |
| Sft2d3 | 0.040 | down | 2.845 | Rattus norvegicus SFT2 domain containing 3 (Sft2d3), mRNA [NM_001108887] |
| LOC498350 | 0.037 | down | 2.756 | Rattus norvegicus similar to testicular haploid expressed gene product isoform 2 (LOC498350), mRNA [NM_001017498] |
| Fam172a | 0.030 | down | 2.721 | Rattus norvegicus family with sequence similarity 172, member A (Fam172a), mRNA [NM_001106401] |
| Cpd | 0.044 | down | 2.662 | Rattus norvegicus carboxypeptidase D (Cpd), mRNA [NM_012836] |
| RGD1562204 | 0.042 | down | 2.633 | PREDICTED: Rattus norvegicus 60S ribosomal protein L9-like (RGD1562204), misc_RNA [XR_146222] |
| Ttc17 | 0.043 | down | 2.611 | Rattus norvegicus tetratricopeptide repeat domain 17 (Ttc17), mRNA [NM_001107752] |
| Pcif1 | 0.027 | down | 2.610 | Rattus norvegicus PDX1 C-terminal inhibiting factor 1 (Pcif1), mRNA [NM_001108605] |
| Usp6nl | 0.037 | down | 2.605 | Rattus norvegicus USP6 N-terminal like (Usp6nl), mRNA [NM_001106120] |
| Degs2 | 0.038 | down | 2.536 | Rattus norvegicus delta(4)-desaturase, sphingolipid 2 (Degs2), mRNA [NM_001017457] |
| Casp2 | 0.038 | down | 2.501 | Rattus norvegicus caspase 2 (Casp2), mRNA [NM_022522] |
| Zfp799 | 0.040 | down | 2.493 | Rattus norvegicus zinc finger protein 799 (Zfp799), mRNA [NM_001009537] |
| Med20 | 0.043 | down | 2.466 | Rattus norvegicus mediator complex subunit 20 (Med20), transcript variant 1, mRNA [NM_001013178] |
| Slc16a1 | 0.043 | down | 2.461 | Rattus norvegicus solute carrier family 16 (monocarboxylate transporter), member 1 (Slc16a1), mRNA [NM_012716] |
| Sesn3 | 0.046 | down | 2.431 | Rattus norvegicus sestrin 3 (Sesn3), mRNA [NM_001108125] |
| LOC317456 | 0.048 | down | 2.419 | Rattus norvegicus hypothetical LOC317456 (LOC317456), mRNA [NM_001047894] |
| Usp25 | 0.044 | down | 2.402 | Rattus norvegicus ubiquitin specific peptidase 25 (Usp25), mRNA [NM_001107114] |
| Prkce | 0.044 | down | 2.386 | Rattus norvegicus protein kinase C, epsilon (Prkce), mRNA [NM_017171] |
| Thumpd2 | 0.037 | down | 2.372 | Rattus norvegicus THUMP domain containing 2 (Thumpd2), transcript variant 2, mRNA [NM_001012108] |
| Cep57l1 | 0.050 | down | 2.370 | Rattus norvegicus centrosomal protein 57-like 1 (Cep57l1), mRNA [NM_001017448] |
| Scai | 0.038 | down | 2.367 | PREDICTED: Rattus norvegicus suppressor of cancer cell invasion (Scai), transcript variant X3, mRNA [XM_006224406] |
| Ppp1r15b | 0.043 | down | 2.358 | Rattus norvegicus protein phosphatase 1, regulatory subunit 15B (Ppp1r15b), mRNA [NM_001107175] |
| Brpf1 | 0.042 | down | 2.357 | Rattus norvegicus bromodomain and PHD finger containing, 1 (Brpf1), mRNA [NM_001191572] |
| RGD1559908 | 0.043 | down | 2.357 | PREDICTED: Rattus norvegicus kinesin-like protein KIF2A-like (RGD1559908), mRNA [XM_006222343] |
| Cep135 | 0.029 | down | 2.357 | Protein Cep135 [Source:UniProtKB/TrEMBL;Acc:D3ZI35] [ENSRNOT00000032156] |
| Hmcn2 | 0.029 | down | 2.351 | PREDICTED: Rattus norvegicus hemicentin 2 (Hmcn2), mRNA [XM_006233927] |
| Ccdc38 | 0.038 | down | 2.342 | PREDICTED: Rattus norvegicus coiled-coil domain containing 38 (Ccdc38), transcript variant X4, mRNA [XM_006226007] |
| Arhgef9 | 0.029 | down | 2.335 | Rattus norvegicus Cdc42 guanine nucleotide exchange factor (GEF) 9 (Arhgef9), mRNA [NM_023957] |
| Taar5 | 0.038 | down | 2.325 | Rattus norvegicus trace amine-associated receptor 5 (Taar5), mRNA [NM_001009650] |
| Mbp | 0.044 | down | 2.325 | Rattus norvegicus myelin basic protein (Mbp), transcript variant 6, mRNA [NM_001025289] |
| Mdfic | 0.043 | down | 2.318 | Rattus norvegicus MyoD family inhibitor domain containing (Mdfic), mRNA [NM_001105668] |
| Syap1 | 0.043 | down | 2.316 | Rattus norvegicus synapse associated protein 1 (Syap1), mRNA [NM_001004253] |
| Sgsm2 | 0.038 | down | 2.310 | Rattus norvegicus small G protein signaling modulator 2 (Sgsm2), mRNA [NM_001107020] |
| Cpne4 | 0.040 | down | 2.282 | Rattus norvegicus copine IV (Cpne4), mRNA [NM_001109003] |
| Oprl1 | 0.038 | down | 2.276 | opiate receptor-like 1 (Oprl1), mRNA [Source:RefSeq mRNA;Acc:NM_031569] [ENSRNOT00000022509] |
| Zfp703 | 0.038 | down | 2.269 | Rattus norvegicus zinc finger protein 703 (Zfp703), mRNA [NM_001109425] |
| Elmo2 | 0.044 | down | 2.252 | Rattus norvegicus engulfment and cell motility 2 (Elmo2), mRNA [NM_001134955] |
| Tchp | 0.043 | down | 2.251 | Rattus norvegicus trichoplein, keratin filament binding (Tchp), mRNA [NM_001191666] |
| Usp12 | 0.043 | down | 2.238 | Rattus norvegicus ubiquitin specific peptidase 12 (Usp12), mRNA [NM_001166576] |
| Gk | 0.043 | down | 2.233 | Rattus norvegicus glycerol kinase (Gk), mRNA [NM_024381] |
| Epm2aip1 | 0.046 | down | 2.230 | Rattus norvegicus EPM2A (laforin) interacting protein 1 (Epm2aip1), mRNA [NM_001271384] |
| Cars | 0.038 | down | 2.225 | Rattus norvegicus cysteinyl-tRNA synthetase (Cars), mRNA [NM_001106319] |
| Chmp4b | 0.037 | down | 2.220 | PREDICTED: Rattus norvegicus charged multivesicular body protein 4B (Chmp4b), mRNA [XM_006224717] |
| Tmem123 | 0.046 | down | 2.213 | Rattus norvegicus transmembrane protein 123 (Tmem123), mRNA [NM_001014205] |
| Zar1l | 0.044 | down | 2.211 | PREDICTED: Rattus norvegicus zygote arrest 1-like (Zar1l), mRNA [XM_002724765] |
| Pptc7 | 0.043 | down | 2.179 | Rattus norvegicus PTC7 protein phosphatase homolog (S. cerevisiae) (Pptc7), mRNA [NM_001107141] |
| Lysmd4 | 0.043 | down | 2.168 | PREDICTED: Rattus norvegicus LysM, putative peptidoglycan-binding, domain containing 4 (Lysmd4), transcript variant X3, mRNA [XM_006223345] |
| Mtmr12 | 0.046 | down | 2.167 | Rattus norvegicus myotubularin related protein 12 (Mtmr12), mRNA [NM_001012077] |
| Rab11fip1 | 0.037 | down | 2.152 | Rattus norvegicus RAB11 family interacting protein 1 (class I) (Rab11fip1), transcript variant 2, mRNA [NM_001197241] |
| Dhrsx | 0.029 | down | 2.150 | Rattus norvegicus dehydrogenase/reductase (SDR family) X-linked (Dhrsx), mRNA [NM_001105914] |
| Rtp3 | 0.027 | down | 2.148 | Rattus norvegicus receptor (chemosensory) transporter protein 3 (Rtp3), mRNA [NM_001108190] |
| Lmnb1 | 0.046 | down | 2.146 | Rattus norvegicus lamin B1 (Lmnb1), mRNA [NM_053905] |
| Phf20l1 | 0.046 | down | 2.140 | PHD finger protein 20-like protein 1 [Source:UniProtKB/Swiss-Prot;Acc:Q4V9H5] [ENSRNOT00000007564] |
| LOC498836 | 0.042 | down | 2.130 | Rattus norvegicus LRRG00125 (LOC498836), mRNA [NM_001047935] |
| Twistnb | 0.037 | down | 2.122 | Rattus norvegicus TWIST neighbor (Twistnb), mRNA [NM_001108707] |
| Amacr | 0.038 | down | 2.115 | Rattus norvegicus alpha-methylacyl-CoA racemase (Amacr), mRNA [NM_012816] |
| Kpna4 | 0.037 | down | 2.112 | Rattus norvegicus karyopherin alpha 4 (importin alpha 3) (Kpna4), mRNA [NM_001014793] |
| Ubtd2 | 0.044 | down | 2.109 | PREDICTED: Rattus norvegicus ubiquitin domain containing 2 (Ubtd2), mRNA [XM_006220990] |
| Clec4e | 0.040 | down | 2.107 | Rattus norvegicus C-type lectin domain family 4, member E (Clec4e), mRNA [NM_001005897] |
| Qser1 | 0.046 | down | 2.094 | Rattus norvegicus glutamine and serine rich 1 (Qser1), mRNA [NM_001139493] |
| Fam8a1 | 0.043 | down | 2.089 | PREDICTED: Rattus norvegicus family with sequence similarity 8, member A1 (Fam8a1), mRNA [XM_001058608] |
| Ssbp2 | 0.038 | down | 2.088 | Protein Ssbp2 [Source:UniProtKB/TrEMBL;Acc:F1M3J3] [ENSRNOT00000021857] |
| Tpd52 | 0.044 | down | 2.082 | Rattus norvegicus tumor protein D52 (Tpd52), mRNA [NM_001106421] |
| Uvssa | 0.043 | down | 2.079 | Rattus norvegicus UV-stimulated scaffold protein A (Uvssa), mRNA [NM_001134558] |
| Rmnd5a | 0.045 | down | 2.077 | PREDICTED: Rattus norvegicus required for meiotic nuclear division 5 homolog A (S. cerevisiae) (Rmnd5a), transcript variant X2, mRNA [XM_232051] |
| Hibch | 0.048 | down | 2.074 | Rattus norvegicus 3-hydroxyisobutyryl-CoA hydrolase (Hibch), mRNA [NM_001013112] |
| Tfb1m | 0.045 | down | 2.070 | Rattus norvegicus transcription factor B1, mitochondrial (Tfb1m), mRNA [NM_181474] |
| Mpp5 | 0.042 | down | 2.067 | PREDICTED: Rattus norvegicus membrane protein, palmitoylated 5 (MAGUK p55 subfamily member 5) (Mpp5), transcript variant X2, mRNA [XM_006240258] |
| Dnaaf2 | 0.048 | down | 2.059 | Rattus norvegicus dynein, axonemal, assembly factor 2 (Dnaaf2), mRNA [NM_001014197] |
| Prpf31 | 0.043 | down | 2.052 | Rattus norvegicus pre-mRNA processing factor 31 (Prpf31), mRNA [NM_001106219] |
| Sec24c | 0.046 | down | 2.049 | Rattus norvegicus SEC24 family member C (Sec24c), mRNA [NM_001109456] |
| Mtrr | 0.046 | down | 2.045 | Rattus norvegicus 5-methyltetrahydrofolate-homocysteine methyltransferase reductase (Mtrr), mRNA [NM_001039003] |
| Commd2 | 0.046 | down | 2.038 | Rattus norvegicus COMM domain containing 2 (Commd2), mRNA [NM_001109503] |
| Cntnap2 | 0.049 | down | 2.033 | PREDICTED: Rattus norvegicus contactin associated protein-like 2 (Cntnap2), partial mRNA [XM_006236412] |
| Gpnmb | 0.044 | down | 2.029 | Rattus norvegicus glycoprotein (transmembrane) nmb (Gpnmb), mRNA [NM_133298] |
| Dpys | 0.038 | down | 2.028 | Rattus norvegicus dihydropyrimidinase (Dpys), mRNA [NM_031705] |
| Fitm2 | 0.046 | down | 2.023 | Rattus norvegicus fat storage-inducing transmembrane protein 2 (Fitm2), mRNA [NM_001107799] |
| Dppa3l1 | 0.046 | down | 2.018 | PREDICTED: Rattus norvegicus developmental pluripotency associated 3-like 1 (Dppa3l1), mRNA [XM_001076514] |
| Aplf | 0.043 | down | 2.013 | Rattus norvegicus aprataxin and PNKP like factor (Aplf), mRNA [NM_001173382] |
| Mcee | 0.043 | down | 2.001 | Rattus norvegicus methylmalonyl CoA epimerase (Mcee), mRNA [NM_001106341] |
