# Supplementary material for: Oral administration of oat beta-glucan preparations of different molecular weight results in regulation of genes connected with immune response in peripheral blood of rats with LPS-induced enteritis
Source: Eur J Nutr. 2018 Oct 4;58(7):2859–73. doi: 10.1007/s00394-018-1838-3 (PMC6769091; doi:10.1007/s00394-018-1838-3)
Supplement: Supplementary file 2 — Supplementary material 2 (DOCX 77 KB) [file 394_2018_1838_MOESM2_ESM.docx]

**Supplementary Table 2.** List of genes differentially expressed in peripheral blood of rats intravenously injected with LPS, with or without dietary sumpplementation with G1 beta-glucan (LPS-G1 vs. LPS-G0). The list presents genes whose expression was significantly changed (p <0.05 and fold change (FC) >2). Genes described in discussion are bolded.

| **GeneSymbol** | **p (Corr)** | **Regulation** | **FC (abs)** | **Description** |
| --- | --- | --- | --- | --- |
| Zfp608 | 0.016 | up | 4.557 | Rattus norvegicus zinc finger protein 608 (Zfp608), mRNA [NM_001107378] |
| Calu | 0.018 | up | 4.476 | Rattus norvegicus calumenin (Calu), transcript variant 2, mRNA [NM_001033898] |
| Serpini2 | 0.018 | up | 4.439 | Rattus norvegicus serpin peptidase inhibitor, clade I, member 2 (Serpini2), mRNA [NM_001134409] |
| Ccdc38 | 0.013 | up | 4.115 | PREDICTED: Rattus norvegicus coiled-coil domain containing 38 (Ccdc38), transcript variant X4, mRNA [XM_006226007] |
| Zfp639 | 0.018 | up | 3.952 | Rattus norvegicus zinc finger protein 639 (Zfp639), mRNA [NM_001080907] |
| Slc39a12 | 0.043 | up | 3.777 | Rattus norvegicus solute carrier family 39 (zinc transporter), member 12 (Slc39a12), mRNA [NM_001106124] |
| Cel | 0.030 | up | 3.579 | Rattus norvegicus carboxyl ester lipase (Cel), mRNA [NM_016997] |
| Spg20 | 0.018 | up | 3.397 | Rattus norvegicus spastic paraplegia 20 (Troyer syndrome) (Spg20), mRNA [NM_001106433] |
| Ankrd27 | 0.040 | up | 3.260 | Rattus norvegicus ankyrin repeat domain 27 (VPS9 domain) (Ankrd27), mRNA [NM_001271264] |
| Brca2 | 0.021 | up | 3.247 | Rattus norvegicus breast cancer 2, early onset (Brca2), mRNA [NM_031542] |
| Fam172a | 0.013 | up | 3.233 | Rattus norvegicus family with sequence similarity 172, member A (Fam172a), mRNA [NM_001106401] |
| Ube2d1 | 0.013 | up | 3.219 | Rattus norvegicus ubiquitin-conjugating enzyme E2D 1 (Ube2d1), mRNA [NM_001108530] |
| RGD1559786 | 0.019 | up | 3.217 | Rattus norvegicus similar to RIKEN cDNA 0610037L13 (RGD1559786), mRNA [NM_001034132] |
| Clcn3 | 0.022 | up | 3.150 | Rattus norvegicus chloride channel, voltage-sensitive 3 (Clcn3), mRNA [NM_053363] |
| Olr479 | 0.043 | up | 3.073 | Rattus norvegicus olfactory receptor 479 (Olr479), mRNA [NM_001000305] |
| Nxpe5 | 0.016 | up | 3.073 | PREDICTED: Rattus norvegicus neurexophilin and PC-esterase domain family, member 5 (Nxpe5), mRNA [XM_221996] |
| Epha5 | 0.044 | up | 3.043 | Rattus norvegicus EPH receptor A5 (Epha5), transcript variant 2, mRNA [NM_024367] |
| P2ry1 | 0.027 | up | 3.031 | Rattus norvegicus purinergic receptor P2Y, G-protein coupled, 1 (P2ry1), mRNA [NM_012800] |
| Dqx1 | 0.032 | up | 3.031 | Rattus norvegicus DEAQ box RNA-dependent ATPase 1 (Dqx1), mRNA [NM_001109407] |
| Fat2 | 0.032 | up | 3.009 | Rattus norvegicus FAT atypical cadherin 2 (Fat2), mRNA [NM_022954] |
| Fbxo3 | 0.019 | up | 2.980 | Rattus norvegicus F-box protein 3 (Fbxo3), mRNA [NM_001109606] |
| LOC102554586 | 0.028 | up | 2.978 | PREDICTED: Rattus norvegicus uncharacterized LOC102554586 (LOC102554586), mRNA [XM_006223522] |
| Nudt13 | 0.043 | up | 2.965 | Rattus norvegicus nudix (nucleoside diphosphate linked moiety X)-type motif 13 (Nudt13), mRNA [NM_001127636] |
| **Mtus1** | **0.018** | **up** | **2.964** | **Rattus norvegicus microtubule associated tumor suppressor 1 (Mtus1), mRNA [NM_178093]** |
| Dmtf1 | 0.020 | up | 2.961 | Rattus norvegicus cyclin D binding myb-like transcription factor 1 (Dmtf1), mRNA [NM_053693] |
| Kcnj5 | 0.032 | up | 2.909 | Rattus norvegicus potassium inwardly-rectifying channel, subfamily J, member 5 (Kcnj5), mRNA [NM_017297] |
| Pde12 | 0.048 | up | 2.905 | phosphodiesterase 12 (Pde12), mRNA [Source:RefSeq mRNA;Acc:NM_001013998] [ENSRNOT00000017800] |
| Rchy1 | 0.032 | up | 2.891 | Rattus norvegicus ring finger and CHY zinc finger domain containing 1, E3 ubiquitin protein ligase (Rchy1), mRNA [NM_001007618] |
| Slc38a6 | 0.019 | up | 2.882 | Rattus norvegicus solute carrier family 38, member 6 (Slc38a6), mRNA [NM_001013099] |
| Kctd20 | 0.040 | up | 2.855 | PREDICTED: Rattus norvegicus potassium channel tetramerization domain containing 20 (Kctd20), transcript variant X3, mRNA [XM_006256150] |
| Sik1 | 0.035 | up | 2.838 | Rattus norvegicus salt-inducible kinase 1 (Sik1), mRNA [NM_021693] |
| LOC685183 | 0.046 | up | 2.834 | PREDICTED: Rattus norvegicus disks large homolog 5-like (LOC685183), transcript variant X1, mRNA [XM_006222691] |
| Ksr1 | 0.016 | up | 2.821 | Rattus norvegicus kinase suppressor of ras 1 (Ksr1), mRNA [NM_001108284] |
| LOC690490 | 0.022 | up | 2.819 | PREDICTED: Rattus norvegicus uncharacterized LOC690490 (LOC690490), mRNA [XM_006227094] |
| Pts | 0.018 | up | 2.815 | Rattus norvegicus 6-pyruvoyl-tetrahydropterin synthase (Pts), mRNA [NM_017220] |
| Lilra5 | 0.030 | up | 2.812 | Rattus norvegicus leukocyte immunoglobulin-like receptor, subfamily A (with TM domain), member 5 (Lilra5), mRNA [NM_001076793] |
| Sqle | 0.013 | up | 2.805 | Rattus norvegicus squalene epoxidase (Sqle), mRNA [NM_017136] |
| Gtf2ird1 | 0.015 | up | 2.796 | Rattus norvegicus GTF2I repeat domain containing 1 (Gtf2ird1), mRNA [NM_001001504] |
| Irgq | 0.037 | up | 2.792 | Rattus norvegicus immunity-related GTPase family, Q (Irgq), mRNA [NM_001135742] |
| Nxpe3 | 0.020 | up | 2.786 | Rattus norvegicus neurexophilin and PC-esterase domain family, member 3 (Nxpe3), mRNA [NM_001109435] |
| Arpc5l | 0.021 | up | 2.783 | Rattus norvegicus actin related protein 2/3 complex, subunit 5-like, mRNA (cDNA clone IMAGE:7377370), complete cds, [BC098820] |
| Lias | 0.030 | up | 2.783 | Rattus norvegicus lipoic acid synthetase (Lias), mRNA [NM_001012037] |
| LOC102550987 | 0.020 | up | 2.781 | PREDICTED: Rattus norvegicus uncharacterized LOC102550987 (LOC102550987), ncRNA [XR_360671] |
| RGD1564324 | 0.016 | up | 2.779 | Rattus norvegicus similar to dehydrogenase/reductase member 2 (RGD1564324), mRNA [NM_001106035] |
| RGD1559908 | 0.018 | up | 2.777 | PREDICTED: Rattus norvegicus kinesin-like protein KIF2A-like (RGD1559908), mRNA [XM_006222343] |
| Prodh | 0.047 | up | 2.777 | Rattus norvegicus proline dehydrogenase (oxidase) 1 (Prodh), nuclear gene encoding mitochondrial protein, mRNA [NM_001135778] |
| Sesn1 | 0.031 | up | 2.777 | Rattus norvegicus sestrin 1 (Sesn1), mRNA [NM_001106396] |
| Ddx25 | 0.019 | up | 2.750 | Rattus norvegicus DEAD (Asp-Glu-Ala-Asp) box helicase 25 (Ddx25), mRNA [NM_031630] |
| Rgl1 | 0.018 | up | 2.735 | Rattus norvegicus ral guanine nucleotide dissociation stimulator,-like 1 (Rgl1), mRNA [NM_001105957] |
| Slc12a1 | 0.025 | up | 2.725 | Rattus norvegicus solute carrier family 12 (sodium/potassium/chloride transporter), member 1 (Slc12a1), transcript variant 1, mRNA [NM_001270617] |
| Fut4 | 0.014 | up | 2.700 | Rattus norvegicus fucosyltransferase 4 (alpha (1,3) fucosyltransferase, myeloid-specific) (Fut4), mRNA [NM_022219] |
| **Atg13** | **0.013** | **up** | **2.698** | **autophagy related 13 (Atg13), mRNA [Source:RefSeq mRNA;Acc:NM_001271212] [ENSRNOT00000023237]** |
| Vamp1 | 0.021 | up | 2.684 | Rattus norvegicus vesicle-associated membrane protein 1 (Vamp1), mRNA [NM_013090] |
| Rgsl1 | 0.047 | up | 2.684 | PREDICTED: Rattus norvegicus regulator of G-protein signaling like 1 (Rgsl1), mRNA [XM_006250025] |
| Cyfip1 | 0.025 | up | 2.673 | Rattus norvegicus cytoplasmic FMR1 interacting protein 1 (Cyfip1), mRNA [NM_001107517] |
| Top1 | 0.043 | up | 2.664 | Rattus norvegicus topoisomerase (DNA) I (Top1), mRNA [NM_022615] |
| Bag2 | 0.029 | up | 2.660 | Rattus norvegicus Bcl2-associated athanogene 2 (Bag2), mRNA [NM_001128195] |
| Kin | 0.020 | up | 2.654 | Rattus norvegicus antigenic determinant of rec-A protein homolog (mouse) (Kin), mRNA [NM_001109529] |
| Zbtb48 | 0.018 | up | 2.654 | Rattus norvegicus zinc finger and BTB domain containing 48 (Zbtb48), mRNA [NM_001013216] |
| Dnajc19 | 0.047 | up | 2.654 | Rattus norvegicus DnaJ (Hsp40) homolog, subfamily C, member 19 (Dnajc19), mRNA [NM_001134640] |
| Kif1c | 0.047 | up | 2.651 | Rattus norvegicus kinesin family member 1C (Kif1c), mRNA [NM_145877] |
| Mbp | 0.021 | up | 2.647 | Rattus norvegicus myelin basic protein (Mbp), transcript variant 6, mRNA [NM_001025289] |
| Crtap | 0.046 | up | 2.628 | Rattus norvegicus cartilage associated protein (Crtap), mRNA [NM_001108785] |
| Elmod2 | 0.036 | up | 2.625 | Rattus norvegicus ELMO/CED-12 domain containing 2 (Elmod2), mRNA [NM_001109506] |
| Cept1 | 0.021 | up | 2.617 | Rattus norvegicus choline/ethanolamine phosphotransferase 1 (Cept1), mRNA [NM_001007699] |
| Atp10d | 0.048 | up | 2.607 | PREDICTED: Rattus norvegicus ATPase, class V, type 10D (Atp10d), mRNA [XM_006250933] |
| Cox16 | 0.026 | up | 2.603 | Rattus norvegicus COX16 cytochrome c oxidase assembly homolog (S, cerevisiae) (Cox16), mRNA [NM_001163153] |
| Cecr5 | 0.030 | up | 2.590 | Rattus norvegicus cat eye syndrome chromosome region, candidate 5 (Cecr5), mRNA [NM_001107884] |
| Zc3h7b | 0.038 | up | 2.585 | Rattus norvegicus zinc finger CCCH-type containing 7B (Zc3h7b), mRNA [NM_001130695] |
| Prr7 | 0.029 | up | 2.585 | Rattus norvegicus proline rich 7 (synaptic) (Prr7), mRNA [NM_001109116] |
| Pknox1 | 0.013 | up | 2.584 | Rattus norvegicus PBX/knotted 1 homeobox 1 (Pknox1), mRNA [NM_001013074] |
| Shprh | 0.045 | up | 2.575 | Rattus norvegicus SNF2 histone linker PHD RING helicase, E3 ubiquitin protein ligase (Shprh), mRNA [NM_001107470] |
| Rnf152 | 0.036 | up | 2.568 | Rattus norvegicus ring finger protein 152 (Rnf152), mRNA [NM_001106305] |
| Ccdc43 | 0.033 | up | 2.566 | Rattus norvegicus coiled-coil domain containing 43 (Ccdc43), mRNA [NM_001100728] |
| Med14 | 0.035 | up | 2.566 | Rattus norvegicus mediator complex subunit 14 (Med14), mRNA [NM_001191727] |
| Phf20l1 | 0.022 | up | 2.563 | PHD finger protein 20-like protein 1 [Source:UniProtKB/Swiss-Prot;Acc:Q4V9H5] [ENSRNOT00000007564] |
| RGD1564095 | 0.036 | up | 2.555 | PREDICTED: Rattus norvegicus 60S acidic ribosomal protein P2-like (RGD1564095), mRNA [XM_006225204] |
| Guf1 | 0.018 | up | 2.546 | Rattus norvegicus GUF1 GTPase homolog (S, cerevisiae) (Guf1), mRNA [NM_001107215] |
| LOC688321 | 0.022 | up | 2.545 | PREDICTED: Rattus norvegicus carbonyl reductase [NADPH] 2-like (LOC688321), mRNA [XM_003750988] |
| She | 0.018 | up | 2.545 | PREDICTED: Rattus norvegicus Src homology 2 domain containing E (She), mRNA [XM_001062249] |
| Rsbn1 | 0.021 | up | 2.534 | Rattus norvegicus round spermatid basic protein 1 (Rsbn1), mRNA [NM_001191710] |
| **Nlrp1b** | **0.016** | **up** | **2.530** | **PREDICTED: Rattus norvegicus NLR family, pyrin domain containing 1B (Nlrp1b), transcript variant X3, mRNA [XM_006220712]** |
| RGD1359127 | 0.022 | up | 2.530 | Rattus norvegicus similar to RIKEN cDNA 2310011J03 (RGD1359127), mRNA [NM_001007657] |
| Fam83c | 0.013 | up | 2.529 | PREDICTED: Rattus norvegicus family with sequence similarity 83, member C (Fam83c), mRNA [XM_001066240] |
| Fam43a | 0.013 | up | 2.522 | Rattus norvegicus family with sequence similarity 43, member A (Fam43a), mRNA [NM_001039002] |
| Nkd2 | 0.018 | up | 2.519 | Rattus norvegicus naked cuticle homolog 2 (Drosophila) (Nkd2), mRNA [NM_001107454] |
| Gmds | 0.036 | up | 2.515 | Rattus norvegicus GDP-mannose 4, 6-dehydratase (Gmds), mRNA [NM_001039606] |
| Cdc25a | 0.029 | up | 2.510 | Rattus norvegicus cell division cycle 25A (Cdc25a), mRNA [NM_133571] |
| Nup153 | 0.038 | up | 2.505 | Rattus norvegicus nucleoporin 153 (Nup153), mRNA [NM_001100470] |
| **Cd40** | **0.029** | **up** | **2.499** | **Rattus norvegicus CD40 molecule, TNF receptor superfamily member 5 (Cd40), mRNA [NM_134360]** |
| Polr3gl | 0.025 | up | 2.499 | Rattus norvegicus polymerase (RNA) III (DNA directed) polypeptide G-like (Polr3gl), mRNA [NM_001109571] |
| LOC102547093 | 0.030 | up | 2.495 | PREDICTED: Rattus norvegicus uncharacterized LOC102547093 (LOC102547093), transcript variant X3, mRNA [XM_006227098] |
| Cetn3 | 0.016 | up | 2.491 | Rattus norvegicus centrin, EF-hand protein, 3 (Cetn3), mRNA [NM_001191842] |
| **Igll1** | **0.014** | **up** | **2.485** | **Rattus norvegicus immunoglobulin lambda-like polypeptide 1 (Igll1), mRNA [NM_001190341]** |
| Hsf2 | 0.015 | up | 2.480 | Rattus norvegicus heat shock transcription factor 2 (Hsf2), mRNA [NM_031694] |
| Lbp | 0.029 | up | 2.478 | Rattus norvegicus lipopolysaccharide binding protein (Lbp), mRNA [NM_017208] |
| Sgk1 | 0.013 | up | 2.474 | Rattus norvegicus serum/glucocorticoid regulated kinase 1 (Sgk1), transcript variant 3, mRNA [NM_019232] |
| Colec12 | 0.014 | up | 2.472 | Rattus norvegicus collectin sub-family member 12 (Colec12), mRNA [NM_001025721] |
| Sft2d3 | 0.030 | up | 2.470 | Rattus norvegicus SFT2 domain containing 3 (Sft2d3), mRNA [NM_001108887] |
| Itih6 | 0.029 | up | 2.467 | PREDICTED: Rattus norvegicus inter-alpha-trypsin inhibitor heavy chain family, member 6 (Itih6), mRNA [XM_003754751] |
| Rab11fip1 | 0.026 | up | 2.463 | Rattus norvegicus RAB11 family interacting protein 1 (class I) (Rab11fip1), transcript variant 2, mRNA [NM_001197241] |
| Zfp319 | 0.023 | up | 2.449 | Rattus norvegicus zinc finger protein 319 (Zfp319), mRNA [NM_001170478] |
| Ggcx | 0.018 | up | 2.448 | Rattus norvegicus gamma-glutamyl carboxylase (Ggcx), mRNA [NM_031756] |
| Riok2 | 0.018 | up | 2.438 | Rattus norvegicus RIO kinase 2 (Riok2), mRNA [NM_001009687] |
| Csnk1g3 | 0.018 | up | 2.433 | Rattus norvegicus casein kinase 1, gamma 3 (Csnk1g3), mRNA [NM_022855] |
| Wdr92 | 0.020 | up | 2.410 | Rattus norvegicus WD repeat domain 92 (Wdr92), mRNA [NM_001127559] |
| Rab26 | 0.033 | up | 2.404 | Rattus norvegicus RAB26, member RAS oncogene family (Rab26), mRNA [NM_133580] |
| Nr3c1 | 0.026 | up | 2.401 | Rattus norvegicus nuclear receptor subfamily 3, group C, member 1 (Nr3c1), mRNA [NM_012576] |
| Emp1 | 0.034 | up | 2.396 | Rattus norvegicus epithelial membrane protein 1 (Emp1), mRNA [NM_012843] |
| Ssbp2 | 0.014 | up | 2.380 | Protein Ssbp2 [Source:UniProtKB/TrEMBL;Acc:F1M3J3] [ENSRNOT00000021857] |
| Tapbpl | 0.016 | up | 2.378 | Rattus norvegicus TAP binding protein-like (Tapbpl), mRNA [NM_001106622] |
| LOC500684 | 0.027 | up | 2.375 | Rattus norvegicus hypothetical protein LOC500684 (LOC500684), mRNA [NM_001047959] |
| Olr1239 | 0.027 | up | 2.373 | Rattus norvegicus olfactory receptor 1239 (Olr1239), mRNA [NM_001000811] |
| **Traf3** | **0.018** | **up** | **2.368** | **Rattus norvegicus Tnf receptor-associated factor 3 (Traf3), mRNA [NM_001108724]** |
| Agl | 0.024 | up | 2.359 | Rattus norvegicus amylo-alpha-1, 6-glucosidase, 4-alpha-glucanotransferase (Agl), mRNA [NM_001108564] |
| RGD1561481 | 0.018 | up | 2.355 | PREDICTED: Rattus norvegicus similar to ubiquitin specific protease 12 (RGD1561481), transcript variant 1, mRNA [XM_001067099] |
| Slc30a1 | 0.046 | up | 2.350 | Rattus norvegicus solute carrier family 30 (zinc transporter), member 1 (Slc30a1), mRNA [NM_022853] |
| Haus3 | 0.018 | up | 2.349 | Rattus norvegicus HAUS augmin-like complex, subunit 3 (Haus3), mRNA [NM_001109412] |
| Luc7l3 | 0.041 | up | 2.348 | PREDICTED: Rattus norvegicus LUC7-like 3 (S, cerevisiae) (Luc7l3), transcript variant X2, mRNA [XM_006247156] |
| Anapc1 | 0.014 | up | 2.347 | Rattus norvegicus anaphase promoting complex subunit 1 (Anapc1), mRNA [NM_001107771] |
| Ankib1 | 0.025 | up | 2.345 | Rattus norvegicus ankyrin repeat and IBR domain containing 1 (Ankib1), mRNA [NM_001134781] |
| LOC288978 | 0.040 | up | 2.343 | Rattus norvegicus hypothetical LOC288978 (LOC288978), mRNA [NM_001134500] |
| Cd2bp2 | 0.035 | up | 2.340 | Rattus norvegicus Cd2 (cytoplasmic tail) binding protein 2 (Cd2bp2), mRNA [NM_001106297] |
| **Fcer1a** | **0.029** | **up** | **2.336** | **Rattus norvegicus Fc fragment of IgE, high affinity I, receptor for; alpha polypeptide (Fcer1a), mRNA [NM_012724]** |
| Slc7a8 | 0.047 | up | 2.336 | Rattus norvegicus solute carrier family 7 (amino acid transporter light chain, L system), member 8 (Slc7a8), mRNA [NM_053442] |
| Tardbp | 0.035 | up | 2.333 | Rattus norvegicus TAR DNA binding protein (Tardbp), mRNA [NM_001011979] |
| Arl11 | 0.031 | up | 2.330 | Rattus norvegicus ADP-ribosylation factor-like 11 (Arl11), mRNA [NM_001013433] |
| Adamtsl3 | 0.041 | up | 2.327 | Rattus norvegicus ADAMTS-like 3 (Adamtsl3), mRNA [NM_001107533] |
| Tgfbr2 | 0.026 | up | 2.321 | Rattus norvegicus transforming growth factor, beta receptor II (Tgfbr2), mRNA [NM_031132] |
| Zmat3 | 0.028 | up | 2.321 | Rattus norvegicus zinc finger, matrin type 3 (Zmat3), mRNA [NM_022548] |
| Hist3h2a | 0.021 | up | 2.321 | Rattus norvegicus histone cluster 3, H2a (Hist3h2a), mRNA [NM_021840] |
| **Atg10** | **0.030** | **up** | **2.319** | **Rattus norvegicus autophagy related 10 (Atg10), mRNA [NM_001109505]** |
| Amacr | 0.039 | up | 2.319 | Rattus norvegicus alpha-methylacyl-CoA racemase (Amacr), mRNA [NM_012816] |
| Gemin7 | 0.024 | up | 2.314 | Rattus norvegicus gem (nuclear organelle) associated protein 7 (Gemin7), mRNA [NM_001109170] |
| RGD1308706 | 0.036 | up | 2.313 | Rattus norvegicus similar to RIKEN cDNA 4921524J17 (RGD1308706), mRNA [NM_001134421] |
| RGD1562629 | 0.049 | up | 2.312 | PREDICTED: Rattus norvegicus neurobeachin-like (RGD1562629), transcript variant X4, mRNA [XM_006224127] |
| Cyp51 | 0.016 | up | 2.311 | Rattus norvegicus cytochrome P450, family 51 (Cyp51), mRNA [NM_012941] |
| Snx22 | 0.029 | up | 2.311 | Rattus norvegicus sorting nexin 22 (Snx22), mRNA [NM_001106832] |
| Bdh1 | 0.018 | up | 2.309 | Rattus norvegicus 3-hydroxybutyrate dehydrogenase, type 1 (Bdh1), mRNA [NM_053995] |
| Rbm28 | 0.036 | up | 2.306 | Rattus norvegicus RNA binding motif protein 28 (Rbm28), mRNA [NM_001107850] |
| RGD1565752 | 0.013 | up | 2.305 | PREDICTED: Rattus norvegicus dnaJ homolog subfamily C member 17-like (RGD1565752), mRNA [XM_001080956] |
| Gprin3 | 0.019 | up | 2.304 | PREDICTED: Rattus norvegicus GPRIN family member 3 (Gprin3), transcript variant X1, mRNA [XM_002726370] |
| Pias3 | 0.019 | up | 2.300 | Rattus norvegicus protein inhibitor of activated STAT, 3 (Pias3), mRNA [NM_031784] |
| Trim32 | 0.033 | up | 2.298 | Rattus norvegicus tripartite motif-containing 32 (Trim32), mRNA [NM_001012103] |
| Twistnb | 0.019 | up | 2.296 | Rattus norvegicus TWIST neighbor (Twistnb), mRNA [NM_001108707] |
| Nkx2-1 | 0.047 | up | 2.296 | Rattus norvegicus NK2 homeobox 1 (Nkx2-1), mRNA [NM_013093] |
| Syap1 | 0.019 | up | 2.289 | Rattus norvegicus synapse associated protein 1 (Syap1), mRNA [NM_001004253] |
| LOC363715 | 0.027 | up | 2.285 | PREDICTED: Rattus norvegicus uncharacterized LOC363715 (LOC363715), mRNA [XM_006221034] |
| Tef | 0.019 | up | 2.283 | Rattus norvegicus thyrotrophic embryonic factor (Tef), mRNA [NM_019194] |
| Dbt | 0.041 | up | 2.280 | Rattus norvegicus dihydrolipoamide branched chain transacylase E2 (Dbt), mRNA [NM_053312] |
| Brpf1 | 0.027 | up | 2.278 | Rattus norvegicus bromodomain and PHD finger containing, 1 (Brpf1), mRNA [NM_001191572] |
| Wdfy3 | 0.044 | up | 2.272 | Rattus norvegicus WD repeat and FYVE domain containing 3 (Wdfy3), mRNA [NM_001170551] |
| Hic2 | 0.047 | up | 2.272 | Rattus norvegicus hypermethylated in cancer 2 (Hic2), mRNA [NM_001105862] |
| Cct6a | 0.030 | up | 2.266 | Rattus norvegicus chaperonin containing Tcp1, subunit 6A (zeta 1) (Cct6a), mRNA [NM_001033684] |
| **Igd** | **0.047** | **up** | **2.266** | **Rattus norvegicus immunoglobulin delta heavy chain secreted form (IgD) mRNA, partial cds, [AY148494]** |
| Pdia5 | 0.039 | up | 2.264 | Rattus norvegicus protein disulfide isomerase family A, member 5 (Pdia5), mRNA [NM_001014125] |
| Ccnt2 | 0.044 | up | 2.263 | Rattus norvegicus cyclin T2 (Ccnt2), mRNA [NM_001107171] |
| Tp53i11 | 0.041 | up | 2.256 | Rattus norvegicus tumor protein p53 inducible protein 11 (Tp53i11), mRNA [NM_001107749] |
| Taf15 | 0.018 | up | 2.256 | Rattus norvegicus TAF15 RNA polymerase II, TATA box binding protein (TBP)-associated factor (Taf15), mRNA [NM_001105824] |
| Cnga1 | 0.044 | up | 2.255 | Rattus norvegicus cyclic nucleotide gated channel alpha 1 (Cnga1), mRNA [NM_053497] |
| Whsc1l1 | 0.013 | up | 2.245 | Rattus norvegicus Wolf-Hirschhorn syndrome candidate 1-like 1 (Whsc1l1), mRNA [NM_001106090] |
| LOC367333 | 0.049 | up | 2.238 | PREDICTED: Rattus norvegicus hypothetical LOC367333 (LOC367333), misc_RNA [XR_146170] |
| Timmdc1 | 0.040 | up | 2.228 | Rattus norvegicus translocase of inner mitochondrial membrane domain containing 1 (Timmdc1), mRNA [NM_001007676] |
| Vax1 | 0.042 | up | 2.217 | Rattus norvegicus ventral anterior homeobox 1 (Vax1), mRNA [NM_022636] |
| Heatr5b | 0.024 | up | 2.213 | Rattus norvegicus HEAT repeat containing 5B (Heatr5b), mRNA [NM_001191064] |
| Snrpc | 0.013 | up | 2.212 | Rattus norvegicus small nuclear ribonucleoprotein polypeptide C (Snrpc), mRNA [NM_001271040] |
| Ccdc117 | 0.017 | up | 2.208 | Rattus norvegicus coiled-coil domain containing 117 (Ccdc117), mRNA [NM_001017502] |
| Mrps27 | 0.020 | up | 2.208 | Rattus norvegicus mitochondrial ribosomal protein S27 (Mrps27), transcript variant 2, mRNA [NM_001108543] |
| LOC304131 | 0.042 | up | 2.205 | Rattus norvegicus similar to C21ORF7 (LOC304131), mRNA [NM_001013979] |
| Zfp770 | 0.020 | up | 2.203 | Protein Zfp770 [Source:UniProtKB/TrEMBL;Acc:F1M076] [ENSRNOT00000067646] |
| Sap30bp | 0.021 | up | 2.200 | Rattus norvegicus SAP30 binding protein (Sap30bp), mRNA [NM_001108305] |
| Wrnip1 | 0.015 | up | 2.197 | Rattus norvegicus Werner helicase interacting protein 1 (Wrnip1), mRNA [NM_172332] |
| Hira | 0.043 | up | 2.194 | Rattus norvegicus histone cell cycle regulator (Hira), mRNA [NM_001135760] |
| Inadl | 0.013 | up | 2.193 | PREDICTED: Rattus norvegicus InaD-like (Drosophila) (Inadl), transcript variant X1, mRNA [XM_006238423] |
| Crebl2 | 0.018 | up | 2.190 | Rattus norvegicus cAMP responsive element binding protein-like 2 (Crebl2), mRNA [NM_001015027] |
| LOC500354 | 0.032 | up | 2.184 | Rattus norvegicus similar to C030030A07Rik protein (LOC500354), mRNA [NM_001037797] |
| Pkhd1l1 | 0.033 | up | 2.182 | Rattus norvegicus polycystic kidney and hepatic disease 1-like 1 (Pkhd1l1), mRNA [NM_001034931] |
| Olr828 | 0.013 | up | 2.173 | Rattus norvegicus olfactory receptor 828 (Olr828), mRNA [NM_001000991] |
| Rfk | 0.034 | up | 2.172 | Rattus norvegicus riboflavin kinase (Rfk), mRNA [NM_001014106] |
| Arhgap24 | 0.032 | up | 2.170 | Rattus norvegicus Rho GTPase activating protein 24 (Arhgap24), mRNA [NM_001012032] |
| Cks1b | 0.046 | up | 2.168 | Rattus norvegicus CDC28 protein kinase regulatory subunit 1B (Cks1b), mRNA [NM_001135749] |
| Ppm1n | 0.018 | up | 2.167 | Rattus norvegicus protein phosphatase, Mg2+/Mn2+ dependent, 1N (Ppm1n), mRNA [NM_001106231] |
| Nup155 | 0.035 | up | 2.159 | Rattus norvegicus nucleoporin 155 (Nup155), mRNA [NM_053952] |
| Mgat4a | 0.020 | up | 2.159 | Rattus norvegicus mannosyl (alpha-1,3-)-glycoprotein beta-1,4-N-acetylglucosaminyltransferase, isozyme A (Mgat4a), transcript variant 1, mRNA [NM_001160155] |
| Efr3b | 0.033 | up | 2.155 | Protein Efr3b [Source:UniProtKB/TrEMBL;Acc:F1LTW9] [ENSRNOT00000039251] |
| Ncoa3 | 0.019 | up | 2.147 | PREDICTED: Rattus norvegicus nuclear receptor coactivator 3 (Ncoa3), mRNA [XM_006224744] |
| Lyc2 | 0.022 | up | 2.145 | Rattus norvegicus lysozyme C type 2 (Lyc2), mRNA [NM_001128494] |
| Inadl | 0.048 | up | 2.145 | InaD-like (Drosophila) [Source:MGI Symbol;Acc:MGI:1277960] [ENSRNOT00000010211] |
| Spetex-2F | 0.016 | up | 2.138 | Rattus norvegicus Spetex-2F protein (Spetex-2F), mRNA [NM_001009968] |
| Sytl4 | 0.018 | up | 2.134 | Rattus norvegicus synaptotagmin-like 4 (Sytl4), mRNA [NM_080410] |
| Lactb2 | 0.036 | up | 2.133 | Rattus norvegicus lactamase, beta 2 (Lactb2), mRNA [NM_001024247] |
| RGD1306227 | 0.046 | up | 2.131 | Rattus norvegicus similar to 4833420G17Rik protein (RGD1306227), mRNA [NM_001014037] |
| LOC690422 | 0.022 | up | 2.128 | RIKEN cDNA 4930427A07 gene [Source:MGI Symbol;Acc:MGI:2144738] [ENSRNOT00000029223] |
| Abhd17c | 0.019 | up | 2.126 | Rattus norvegicus abhydrolase domain containing 17C (Abhd17c), mRNA [NM_001100736] |
| Tp53rk | 0.030 | up | 2.126 | Rattus norvegicus TP53 regulating kinase (Tp53rk), mRNA [NM_001108606] |
| Gngt1 | 0.020 | up | 2.122 | Rattus norvegicus guanine nucleotide binding protein (G protein), gamma transducing activity polypeptide 1 (Gngt1), mRNA [NM_001135777] |
| Tmem55b | 0.018 | up | 2.122 | Rattus norvegicus transmembrane protein 55B (Tmem55b), mRNA [NM_001014233] |
| Hoxa9l | 0.026 | up | 2.120 | PREDICTED: Rattus norvegicus homeobox A9-like (Hoxa9l), transcript variant X1, mRNA [XM_001057018] |
| Pikfyve | 0.036 | up | 2.117 | Protein Pikfyve; Similar to phosphatidylinositol-3-phosphate/phosphatidylinositol 5-kinase, type III isoform 2 [Source:UniProtKB/TrEMBL;Acc:D3ZT14] [ENSRNOT00000020447] |
| Ppfibp1 | 0.038 | up | 2.114 | Rattus norvegicus PTPRF interacting protein, binding protein 1 (liprin beta 1) (Ppfibp1), mRNA [NM_001107896] |
| Akap9 | 0.032 | up | 2.112 | Rattus norvegicus A kinase (PRKA) anchor protein 9 (Akap9), mRNA [NM_001037093] |
| Ccdc88a | 0.028 | up | 2.107 | PREDICTED: Rattus norvegicus coiled coil domain containing 88A (Ccdc88a), transcript variant X2, mRNA [XM_006221877] |
| Tmem81 | 0.046 | up | 2.106 | Rattus norvegicus transmembrane protein 81 (Tmem81), mRNA [NM_001017490] |
| Taok1 | 0.022 | up | 2.102 | Rattus norvegicus TAO kinase 1 (Taok1), mRNA [NM_173327] |
| Eid3 | 0.036 | up | 2.101 | Rattus norvegicus EP300 interacting inhibitor of differentiation 3 (Eid3), mRNA [NM_001044304] |
| Cars | 0.019 | up | 2.097 | Rattus norvegicus cysteinyl-tRNA synthetase (Cars), mRNA [NM_001106319] |
| Tmed3 | 0.018 | up | 2.096 | Rattus norvegicus transmembrane emp24 protein transport domain containing 3 (Tmed3), mRNA [NM_001004249] |
| Dppa3l1 | 0.018 | up | 2.096 | PREDICTED: Rattus norvegicus developmental pluripotency associated 3-like 1 (Dppa3l1), mRNA [XM_001076514] |
| Heatr6 | 0.021 | up | 2.095 | Rattus norvegicus HEAT repeat containing 6 (Heatr6), mRNA [NM_001079897] |
| Exo5 | 0.044 | up | 2.092 | Rattus norvegicus exonuclease 5 (Exo5), mRNA [NM_001107973] |
| LOC100911339 | 0.022 | up | 2.091 | PREDICTED: Rattus norvegicus uncharacterized LOC100911339 (LOC100911339), misc_RNA [XR_146006] |
| Klhl14 | 0.018 | up | 2.088 | Rattus norvegicus kelch-like family member 14 (Klhl14), mRNA [NM_001108885] |
| Inppl1 | 0.042 | up | 2.081 | Rattus norvegicus inositol polyphosphate phosphatase-like 1 (Inppl1), transcript variant 1, mRNA [NM_022944] |
| Lpar3 | 0.044 | up | 2.079 | Rattus norvegicus lysophosphatidic acid receptor 3 (Lpar3), mRNA [NM_023969] |
| Tspan15 | 0.043 | up | 2.076 | Rattus norvegicus tetraspanin 15 (Tspan15), mRNA [NM_001115032] |
| Polb | 0.014 | up | 2.074 | polymerase (DNA directed), beta (Polb), mRNA [Source:RefSeq mRNA;Acc:NM_017141] [ENSRNOT00000026039] |
| Srpk3 | 0.016 | up | 2.074 | Rattus norvegicus SRSF protein kinase 3 (Srpk3), mRNA [NM_184045] |
| Marf1 | 0.018 | up | 2.073 | Rattus norvegicus meiosis arrest female 1 (Marf1), mRNA [NM_133421] |
| Mpeg1 | 0.036 | up | 2.072 | Rattus norvegicus macrophage expressed 1 (Mpeg1), mRNA [NM_022617] |
| Cc2d1b | 0.040 | up | 2.071 | Rattus norvegicus coiled-coil and C2 domain containing 1B (Cc2d1b), mRNA [NM_001270984] |
| Catsper3 | 0.042 | up | 2.071 | Rattus norvegicus cation channel, sperm associated 3 (Catsper3), mRNA [NM_001106101] |
| LOC102548917 | 0.044 | up | 2.070 | PREDICTED: Rattus norvegicus zinc finger protein 844-like (LOC102548917), transcript variant X1, mRNA [XM_006222164] |
| Chuk | 0.022 | up | 2.069 | Rattus norvegicus conserved helix-loop-helix ubiquitous kinase (Chuk), mRNA [NM_001107588] |
| Ddhd2 | 0.034 | up | 2.068 | PREDICTED: Rattus norvegicus DDHD domain containing 2 (Ddhd2), transcript variant X4, mRNA [XM_006253339] |
| Tmem185a | 0.018 | up | 2.067 | Rattus norvegicus transmembrane protein 185A (Tmem185a), mRNA [NM_001135712] |
| Eif1b | 0.018 | up | 2.066 | Rattus norvegicus eukaryotic translation initiation factor 1B (Eif1b), mRNA [NM_001106867] |
| Sertm1 | 0.031 | up | 2.066 | Rattus norvegicus serine-rich and transmembrane domain containing 1 (Sertm1), mRNA [NM_001109580] |
| LOC691277 | 0.018 | up | 2.063 | Rattus norvegicus similar to Robo-1 (LOC691277), mRNA [NM_001109633] |
| Wtap | 0.035 | up | 2.061 | Rattus norvegicus Wilms tumor 1 associated protein (Wtap), transcript variant 1, mRNA [NM_001113542] |
| RGD1305202 | 0.018 | up | 2.057 | PREDICTED: Rattus norvegicus cerebral cavernous malformations 2 protein-like (RGD1305202), mRNA [XM_003749594] |
| Adal | 0.036 | up | 2.054 | Rattus norvegicus adenosine deaminase-like (Adal), mRNA [NM_001014047] |
| Ube2k | 0.019 | up | 2.053 | Rattus norvegicus ubiquitin-conjugating enzyme E2K (Ube2k), mRNA [NM_001106006] |
| Swsap1 | 0.036 | up | 2.052 | Rattus norvegicus SWIM-type zinc finger 7 associated protein 1 (Swsap1), mRNA [NM_001108755] |
| LOC681325 | 0.021 | up | 2.051 | Rattus norvegicus hypothetical protein LOC681325 (LOC681325), mRNA [NM_001109439] |
| Nrip3 | 0.031 | up | 2.051 | Rattus norvegicus nuclear receptor interacting protein 3 (Nrip3), mRNA [NM_001108498] |
| Pip5k1a | 0.029 | up | 2.047 | Rattus norvegicus phosphatidylinositol-4-phosphate 5-kinase, type 1, alpha (Pip5k1a), mRNA [NM_001042621] |
| Papd7 | 0.018 | up | 2.046 | Rattus norvegicus PAP associated domain containing 7 (Papd7), mRNA [NM_001107333] |
| Slc52a2 | 0.034 | up | 2.046 | Rattus norvegicus solute carrier family 52, riboflavin transporter, member 2 (Slc52a2), mRNA [NM_001109670] |
| Mccc2 | 0.040 | up | 2.044 | Rattus norvegicus methylcrotonoyl-CoA carboxylase 2 (beta) (Mccc2), mRNA [NM_001012177] |
| Ppp2r1b | 0.016 | up | 2.044 | Rattus norvegicus protein phosphatase 2, regulatory subunit A, beta (Ppp2r1b), mRNA [NM_001025418] |
| Snx11 | 0.028 | up | 2.043 | Rattus norvegicus sorting nexin 11 (Snx11), transcript variant 1, mRNA [NM_001012012] |
| Lgals7 | 0.018 | up | 2.041 | Rattus norvegicus lectin, galactoside-binding, soluble, 7 (Lgals7), mRNA [NM_022582] |
| Epm2aip1 | 0.026 | up | 2.040 | Rattus norvegicus EPM2A (laforin) interacting protein 1 (Epm2aip1), mRNA [NM_001271384] |
| Abcf2 | 0.030 | up | 2.039 | Rattus norvegicus ATP-binding cassette, subfamily F (GCN20), member 2 (Abcf2), mRNA [NM_001109666] |
| Rbm48 | 0.018 | up | 2.038 | Rattus norvegicus RNA binding motif protein 48 (Rbm48), mRNA [NM_001024246] |
| Golga1 | 0.029 | up | 2.037 | Rattus norvegicus golgin A1 (Golga1), mRNA [NM_001107842] |
| Kif16b | 0.036 | up | 2.035 | Rattus norvegicus kinesin family member 16B (Kif16b), mRNA [NM_001107783] |
| Ppil3 | 0.018 | up | 2.033 | Rattus norvegicus peptidylprolyl isomerase (cyclophilin)-like 3 (Ppil3), mRNA [NM_175707] |
| Scai | 0.019 | up | 2.033 | PREDICTED: Rattus norvegicus suppressor of cancer cell invasion (Scai), transcript variant X3, mRNA [XM_006224406] |
| Plekhg1 | 0.045 | up | 2.033 | Rattus norvegicus pleckstrin homology domain containing, family G (with RhoGef domain) member 1 (Plekhg1), mRNA [NM_001190999] |
| Lpar2 | 0.042 | up | 2.032 | Rattus norvegicus lysophosphatidic acid receptor 2 (Lpar2), mRNA [NM_001109109] |
| Fggy | 0.019 | up | 2.032 | Rattus norvegicus FGGY carbohydrate kinase domain containing (Fggy), mRNA [NM_001013932] |
| Fcer2 | 0.021 | up | 2.031 | Rattus norvegicus Fc fragment of IgE, low affinity II, receptor for (CD23) (Fcer2), transcript variant 2, mRNA [NM_133550] |
| Agpat3 | 0.024 | up | 2.030 | Rattus norvegicus 1-acylglycerol-3-phosphate O-acyltransferase 3 (Agpat3), mRNA [NM_001106378] |
| Psat1 | 0.029 | up | 2.029 | Rattus norvegicus phosphoserine aminotransferase 1 (Psat1), mRNA [NM_198738] |
| Ppm1d | 0.048 | up | 2.027 | Rattus norvegicus protein phosphatase, Mg2+/Mn2+ dependent, 1D (Ppm1d), mRNA [NM_001105825] |
| Trps1 | 0.036 | up | 2.024 | Rattus norvegicus trichorhinophalangeal syndrome I (Trps1), mRNA [NM_001134837] |
| Cc2d2a | 0.026 | up | 2.024 | coiled-coil and C2 domain containing 2A [Source:MGI Symbol;Acc:MGI:1924487] [ENSRNOT00000006968] |
| Tnnt3 | 0.040 | up | 2.023 | Rattus norvegicus troponin T type 3 (skeletal, fast) (Tnnt3), transcript variant 1, mRNA [NM_001270665] |
| Lgals8 | 0.024 | up | 2.023 | Rattus norvegicus lectin, galactoside-binding, soluble, 8 (Lgals8), mRNA [NM_053862] |
| Mfsd9 | 0.018 | up | 2.020 | Rattus norvegicus major facilitator superfamily domain containing 9 (Mfsd9), mRNA [NM_001108215] |
| Mpp5 | 0.023 | up | 2.020 | PREDICTED: Rattus norvegicus membrane protein, palmitoylated 5 (MAGUK p55 subfamily member 5) (Mpp5), transcript variant X2, mRNA [XM_006240258] |
| LOC691670 | 0.039 | up | 2.018 | Rattus norvegicus similar to natural killer cell protease 7 (LOC691670), mRNA [NM_001109646] |
| Pwwp2a | 0.048 | up | 2.018 | PREDICTED: Rattus norvegicus PWWP domain containing 2A (Pwwp2a), transcript variant X1, mRNA [XM_006246135] |
| Armc10 | 0.021 | up | 2.016 | Rattus norvegicus armadillo repeat containing 10 (Armc10), mRNA [NM_001106576] |
| Pptc7 | 0.018 | up | 2.014 | Rattus norvegicus PTC7 protein phosphatase homolog (S, cerevisiae) (Pptc7), mRNA [NM_001107141] |
| Lrp4 | 0.032 | up | 2.013 | Rattus norvegicus low density lipoprotein receptor-related protein 4 (Lrp4), mRNA [NM_031322] |
| Slc25a22 | 0.049 | up | 2.013 | Rattus norvegicus solute carrier family 25 (mitochondrial carrier, glutamate), member 22 (Slc25a22), mRNA [NM_001014027] |
| Angel2 | 0.030 | up | 2.012 | Rattus norvegicus angel homolog 2 (Drosophila) (Angel2), mRNA [NM_001135119] |
| Rlim | 0.024 | up | 2.008 | Rattus norvegicus ring finger protein, LIM domain interacting (Rlim), mRNA [NM_001024892] |
| Cd40 | 0.018 | up | 2.008 | Rattus norvegicus CD40 molecule, TNF receptor superfamily member 5 (Cd40), mRNA [NM_134360] |
| Slc7a6os | 0.029 | up | 2.003 | Rattus norvegicus solute carrier family 7, member 6 opposite strand (Slc7a6os), mRNA [NM_139328] |
| Etv5 | 0.038 | up | 2.003 | Rattus norvegicus ets variant 5 (Etv5), mRNA [NM_001107082] |
| Lhpp | 0.018 | up | 2.003 | Rattus norvegicus phospholysine phosphohistidine inorganic pyrophosphate phosphatase (Lhpp), mRNA [NM_001009706] |
| Olr1592 | 0.045 | up | 2.001 | Rattus norvegicus olfactory receptor 1592 (Olr1592), mRNA [NM_001000084] |
| Serp2 | 0.014 | down | 12.594 | Rattus norvegicus stress-associated endoplasmic reticulum protein family member 2 (Serp2), mRNA [NM_001109104] |
| Slc27a2 | 0.021 | down | 5.709 | Rattus norvegicus solute carrier family 27 (fatty acid transporter), member 2 (Slc27a2), mRNA [NM_031736] |
| Ube2c | 0.013 | down | 5.648 | Rattus norvegicus ubiquitin-conjugating enzyme E2C (Ube2c), mRNA [NM_001106542] |
| Frem3 | 0.013 | down | 5.053 | Rattus norvegicus FRAS1 related extracellular matrix 3 (Frem3), mRNA [NM_001191699] |
| Tmem121 | 0.032 | down | 3.687 | PREDICTED: Rattus norvegicus transmembrane protein 121 (Tmem121), transcript variant X1, mRNA [XM_001079243] |
| Kif18b | 0.041 | down | 3.531 | Rattus norvegicus kinesin family member 18B (Kif18b), mRNA [NM_001039019] |
| RGD1310495 | 0.013 | down | 3.529 | PREDICTED: Rattus norvegicus sodium-dependent glucose transporter 1A-like (RGD1310495), mRNA [XM_006223921] |
| LOC102547119 | 0.025 | down | 3.387 | PREDICTED: Rattus norvegicus spermatogenesis-associated protein 31C2-like (LOC102547119), mRNA [XM_006257130] |
| Egr3 | 0.025 | down | 3.331 | Rattus norvegicus early growth response 3 (Egr3), mRNA [NM_017086] |
| Olr1406 | 0.029 | down | 3.281 | Rattus norvegicus olfactory receptor 1406 (Olr1406), mRNA [NM_001000786] |
| Tpx2 | 0.045 | down | 3.203 | Rattus norvegicus TPX2, microtubule-associated (Tpx2), mRNA [NM_001107790] |
| Tspan18 | 0.026 | down | 3.187 | Rattus norvegicus tetraspanin 18 (Tspan18), mRNA [NM_001107750] |
| Kifc1 | 0.021 | down | 3.148 | Rattus norvegicus kinesin family member C1 (Kifc1), mRNA [NM_001005878] |
| B3gat1 | 0.018 | down | 3.078 | PREDICTED: Rattus norvegicus beta-1,3-glucuronyltransferase 1 (glucuronosyltransferase P) (B3gat1), transcript variant X1, mRNA [XM_006242733] |
| Dmtn | 0.021 | down | 3.041 | Rattus norvegicus dematin actin binding protein (Dmtn), mRNA [NM_001108385] |
| Nprl3 | 0.047 | down | 3.040 | Rattus norvegicus nitrogen permease regulator-like 3 (S, cerevisiae) (Nprl3), mRNA [NM_001034936] |
| Sfxn2 | 0.018 | down | 3.039 | Rattus norvegicus sideroflexin 2 (Sfxn2), mRNA [NM_001013072] |
| Olr157 | 0.018 | down | 2.961 | Rattus norvegicus olfactory receptor 157 (Olr157), mRNA [NM_001000169] |
| Ckap2l | 0.022 | down | 2.959 | PREDICTED: Rattus norvegicus cytoskeleton associated protein 2-like (Ckap2l), transcript variant X2, mRNA [XM_006224611] |
| Tro | 0.023 | down | 2.940 | trophinin [Source:MGI Symbol;Acc:MGI:1928994] [ENSRNOT00000071639] |
| RGD1561017 | 0.039 | down | 2.901 | PREDICTED: Rattus norvegicus ral guanine nucleotide dissociation stimulator-like (RGD1561017), misc_RNA [XR_338617] |
| Rrm2 | 0.013 | down | 2.891 | Rattus norvegicus ribonucleotide reductase M2 (Rrm2), mRNA [NM_001025740] |
| Bpifa2 | 0.023 | down | 2.879 | Rattus norvegicus BPI fold containing family A, member 2 (Bpifa2), mRNA [NM_052808] |
| Gpt2 | 0.018 | down | 2.853 | glutamic pyruvate transaminase (alanine aminotransferase) 2 (Gpt2), mRNA [Source:RefSeq mRNA;Acc:NM_001012057] [ENSRNOT00000071992] |
| Stfa2l1 | 0.025 | down | 2.839 | Rattus norvegicus stefin A2-like 1 (Stfa2l1), mRNA [NM_001004129] |
| LOC498236 | 0.047 | down | 2.834 | Rattus norvegicus LRRGT00186 (LOC498236), mRNA [NM_001047926] |
| **Kcng2** | **0.013** | **down** | **2.829** | **Rattus norvegicus potassium voltage-gated channel, subfamily G, member 2 (Kcng2), mRNA [NM_001107372]** |
| LOC688874 | 0.044 | down | 2.761 | PREDICTED: Rattus norvegicus NEDD4-binding protein 1-like (LOC688874), mRNA [XM_006224825] |
| Zzz3 | 0.033 | down | 2.750 | Rattus norvegicus zinc finger, ZZ-type containing 3 (Zzz3), mRNA [NM_001134549] |
| Clvs1 | 0.036 | down | 2.747 | Rattus norvegicus clavesin 1 (Clvs1), mRNA [NM_001108969] |
| LOC100910984 | 0.013 | down | 2.735 | family with sequence similarity 150, member A [Source:MGI Symbol;Acc:MGI:3645495] [ENSRNOT00000073694] |
| LOC498155 | 0.018 | down | 2.733 | Rattus norvegicus LRRGT00167 (LOC498155), mRNA [NM_001047924] |
| Zfp287 | 0.018 | down | 2.702 | Rattus norvegicus zinc finger protein 287 (Zfp287), mRNA [NM_001107008] |
| Abca17 | 0.019 | down | 2.693 | Rattus norvegicus ATP-binding cassette, subfamily A (ABC1), member 17 (Abca17), mRNA [NM_001031637] |
| Tmem182 | 0.017 | down | 2.689 | Rattus norvegicus transmembrane protein 182 (Tmem182), mRNA [NM_001109305] |
| Dmrtc1b | 0.036 | down | 2.689 | Protein Dmrtc1b [Source:UniProtKB/TrEMBL;Acc:F1LU90] [ENSRNOT00000029245] |
| Bnipl | 0.027 | down | 2.628 | Rattus norvegicus BCL2/adenovirus E1B 19kD interacting protein like (Bnipl), mRNA [NM_001128187] |
| LOC502358 | 0.025 | down | 2.625 | PREDICTED: Rattus norvegicus hemoglobin subunit epsilon-like (LOC502358), mRNA [XM_003748943] |
| LOC102547612 | 0.030 | down | 2.620 | PREDICTED: Rattus norvegicus uncharacterized LOC102547612 (LOC102547612), ncRNA [XR_344811] |
| Maff | 0.018 | down | 2.611 | Rattus norvegicus v-maf avian musculoaponeurotic fibrosarcoma oncogene homolog F (Maff), mRNA [NM_001130573] |
| Usp17l5 | 0.036 | down | 2.608 | PREDICTED: Rattus norvegicus ubiquitin specific peptidase 17-like 5 (Usp17l5), mRNA [XM_001074163] |
| Clybl | 0.019 | down | 2.602 | Rattus norvegicus citrate lyase beta like (Clybl), mRNA [NM_001100685] |
| Atp6v1g2 | 0.036 | down | 2.588 | Rattus norvegicus ATPase, H+ transporting, lysosomal V1 subunit G2 (Atp6v1g2), mRNA [NM_212490] |
| Cdca3 | 0.018 | down | 2.583 | Rattus norvegicus cell division cycle associated 3 (Cdca3), mRNA [NM_001007648] |
| Slc26a9 | 0.042 | down | 2.577 | Rattus norvegicus solute carrier family 26 (anion exchanger), member 9 (Slc26a9), mRNA [NM_001107172] |
| Glyatl3 | 0.018 | down | 2.565 | Rattus norvegicus glycine-N-acyltransferase-like 3 (Glyatl3), mRNA [NM_001145062] |
| RGD1563222 | 0.013 | down | 2.561 | Rattus norvegicus similar to RIKEN cDNA A930018P22 (RGD1563222), mRNA [NM_001108585] |
| Tyms | 0.018 | down | 2.556 | Rattus norvegicus thymidylate synthetase (Tyms), mRNA [NM_019179] |
| Tm4sf4 | 0.023 | down | 2.547 | Rattus norvegicus transmembrane 4 L six family member 4 (Tm4sf4), mRNA [NM_053785] |
| Kiss1r | 0.015 | down | 2.540 | Rattus norvegicus KISS1 receptor (Kiss1r), mRNA [NM_023992] |
| RGD1563451 | 0.036 | down | 2.526 | PREDICTED: Rattus norvegicus TD and POZ domain-containing protein 2-like (RGD1563451), mRNA [XM_003753628] |
| Olr1486 | 0.032 | down | 2.517 | Rattus norvegicus olfactory receptor 1486 (Olr1486), mRNA [NM_001000720] |
| RGD1561277 | 0.031 | down | 2.500 | PREDICTED: Rattus norvegicus RGD1561277 (RGD1561277), mRNA [XM_006220995] |
| Zfp496 | 0.035 | down | 2.499 | Rattus norvegicus zinc finger protein 496 (Zfp496), mRNA [NM_001271396] |
| Bmp8b | 0.039 | down | 2.495 | PREDICTED: Rattus norvegicus bone morphogenetic protein 8b (Bmp8b), transcript variant X2, mRNA [XM_006238834] |
| Pfkfb3 | 0.018 | down | 2.488 | Rattus norvegicus 6-phosphofructo-2-kinase/fructose-2,6-biphosphatase 3 (Pfkfb3), mRNA [NM_057135] |
| Slmo1 | 0.018 | down | 2.478 | Rattus norvegicus slowmo homolog 1 (Drosophila) (Slmo1), mRNA [NM_001109570] |
| Tspan11 | 0.016 | down | 2.477 | Rattus norvegicus tetraspanin 11 (Tspan11), mRNA [NM_001024262] |
| Wdr86 | 0.044 | down | 2.473 | Rattus norvegicus WD repeat domain 86 (Wdr86), mRNA [NM_001110489] |
| Olr311 | 0.018 | down | 2.464 | Rattus norvegicus olfactory receptor 311 (Olr311), mRNA [NM_001000555] |
| Chac2 | 0.020 | down | 2.460 | Rattus norvegicus ChaC, cation transport regulator homolog 2 (E, coli) (Chac2), mRNA [NM_001025016] |
| Cmtm2a | 0.022 | down | 2.433 | Rattus norvegicus CKLF-like MARVEL transmembrane domain containing 2A (Cmtm2a), mRNA [NM_001013142] |
| Evpl | 0.023 | down | 2.431 | Rattus norvegicus envoplakin (Evpl), mRNA [NM_001107066] |
| RGD1562485 | 0.025 | down | 2.429 | Rattus norvegicus similar to RIKEN cDNA 4921511C20 gene (RGD1562485), mRNA [NM_001106966] |
| Chac2 | 0.018 | down | 2.412 | Rattus norvegicus ChaC, cation transport regulator homolog 2 (E, coli) (Chac2), mRNA [NM_001025016] |
| Wasf3 | 0.020 | down | 2.402 | PREDICTED: Rattus norvegicus WAS protein family, member 3 (Wasf3), mRNA [XM_006248846] |
| Slc24a5 | 0.018 | down | 2.402 | Rattus norvegicus solute carrier family 24 (sodium/potassium/calcium exchanger), member 5 (Slc24a5), mRNA [NM_001107769] |
| Tnfrsf13b | 0.036 | down | 2.398 | Protein Tnfrsf13b [Source:UniProtKB/TrEMBL;Acc:F1LX35] [ENSRNOT00000003139] |
| Lrrc20 | 0.019 | down | 2.396 | Rattus norvegicus leucine rich repeat containing 20 (Lrrc20), mRNA [NM_001109171] |
| LOC685125 | 0.018 | down | 2.392 | PREDICTED: Rattus norvegicus disks large homolog 5-like (LOC685125), partial mRNA [XM_006255360] |
| Adc | 0.031 | down | 2.388 | Rattus norvegicus arginine decarboxylase (Adc), mRNA [NM_001014261] |
| Icmt | 0.016 | down | 2.385 | Rattus norvegicus isoprenylcysteine carboxyl methyltransferase (Icmt), mRNA [NM_133310] |
| Mmgt2 | 0.021 | down | 2.382 | Rattus norvegicus membrane magnesium transporter 2 (Mmgt2), mRNA [NM_001013967] |
| Cst12 | 0.018 | down | 2.380 | Rattus norvegicus cystatin 12 (Cst12), mRNA [NM_153734] |
| RGD1564836 | 0.029 | down | 2.373 | Protein RGD1564836 [Source:UniProtKB/TrEMBL;Acc:D3ZAX1] [ENSRNOT00000038940] |
| Nbl1 | 0.026 | down | 2.370 | Rattus norvegicus neuroblastoma 1, DAN family BMP antagonist (Nbl1), mRNA [NM_031609] |
| Chst15 | 0.030 | down | 2.370 | PREDICTED: Rattus norvegicus carbohydrate (N-acetylgalactosamine 4-sulfate 6-O) sulfotransferase 15 (Chst15), transcript variant X1, mRNA [XM_006230430] |
| Hs3st3a1 | 0.029 | down | 2.370 | Rattus norvegicus heparan sulfate (glucosamine) 3-O-sulfotransferase 3A1 (Hs3st3a1), mRNA [NM_001172067] |
| LOC686031 | 0.043 | down | 2.368 | Rattus norvegicus hypothetical protein LOC686031 (LOC686031), mRNA [NM_001109490] |
| Dsg4 | 0.033 | down | 2.366 | Rattus norvegicus desmoglein 4 (Dsg4), mRNA [NM_199490] |
| Rtp1 | 0.021 | down | 2.359 | Rattus norvegicus receptor (chemosensory) transporter protein 1 (Rtp1), mRNA [NM_001105870] |
| LOC691920 | 0.039 | down | 2.358 | START domain containing 9 [Source:MGI Symbol;Acc:MGI:3045258] [ENSRNOT00000048141] |
| Wfdc1 | 0.014 | down | 2.355 | Rattus norvegicus WAP four-disulfide core domain 1 (Wfdc1), mRNA [NM_133581] |
| RGD1560608 | 0.013 | down | 2.355 | Rattus norvegicus similar to novel protein (RGD1560608), mRNA [NM_001109280] |
| Pced1b | 0.019 | down | 2.348 | Rattus norvegicus PC-esterase domain containing 1B (Pced1b), mRNA [NM_001039454] |
| **RGD1560650** | **0.025** | **down** | **2.343** | **PREDICTED: Rattus norvegicus granzyme C-like (LOC691695), mRNA [XM_006221943]** |
| Slc6a9 | 0.021 | down | 2.343 | Rattus norvegicus solute carrier family 6 (neurotransmitter transporter, glycine), member 9 (Slc6a9), mRNA [NM_053818] |
| LOC689039 | 0.015 | down | 2.330 | PREDICTED: Rattus norvegicus hypothetical protein LOC689039 (LOC689039), mRNA [XM_001069291] |
| Spag5 | 0.019 | down | 2.322 | Rattus norvegicus sperm associated antigen 5 (Spag5), mRNA [NM_001044224] |
| LOC102548134 | 0.018 | down | 2.322 | PREDICTED: Rattus norvegicus uncharacterized LOC102548134 (LOC102548134), transcript variant X2, ncRNA [XR_349135] |
| Hapln4 | 0.029 | down | 2.319 | Rattus norvegicus hyaluronan and proteoglycan link protein 4 (Hapln4), mRNA [NM_001108398] |
| Phox2b | 0.036 | down | 2.319 | PREDICTED: Rattus norvegicus paired-like homeobox 2b (Phox2b), mRNA [XM_006221760] |
| LOC685888 | 0.029 | down | 2.318 | RIKEN cDNA 1110058L19 gene [Source:MGI Symbol;Acc:MGI:1915252] [ENSRNOT00000035033] |
| Trim10 | 0.021 | down | 2.318 | Rattus norvegicus tripartite motif-containing 10 (Trim10), mRNA [NM_001009176] |
| Vsx1 | 0.049 | down | 2.315 | Rattus norvegicus visual system homeobox 1 (Vsx1), mRNA [NM_001109546] |
| Kel | 0.039 | down | 2.308 | Rattus norvegicus Kell blood group, metallo-endopeptidase (Kel), mRNA [NM_001191611] |
| Vom1r8 | 0.024 | down | 2.305 | Rattus norvegicus vomeronasal 1 receptor 8 (Vom1r8), mRNA [NM_001008905] |
| Krtap3-1 | 0.036 | down | 2.304 | PREDICTED: Rattus norvegicus keratin associated protein 3-1 (Krtap3-1), mRNA [XM_001055567] |
| Gpr52 | 0.029 | down | 2.304 | Rattus norvegicus G protein-coupled receptor 52 (Gpr52), mRNA [NM_001289935] |
| RGD1563155 | 0.029 | down | 2.302 | Rattus norvegicus similar to RIKEN cDNA 1700054O13 (RGD1563155), mRNA [NM_001134598] |
| Ces1e | 0.014 | down | 2.295 | Rattus norvegicus carboxylesterase 1E (Ces1e), mRNA [NM_031565] |
| **Scn7a** | **0.019** | **down** | **2.283** | **Rattus norvegicus sodium channel, voltage-gated, type VII, alpha (Scn7a), mRNA [NM_031686]** |
| Sult4a1 | 0.036 | down | 2.279 | Rattus norvegicus sulfotransferase family 4A, member 1 (Sult4a1), mRNA [NM_031641] |
| Itga7 | 0.040 | down | 2.277 | Rattus norvegicus integrin, alpha 7 (Itga7), mRNA [NM_030842] |
| Rnf40 | 0.030 | down | 2.276 | Rattus norvegicus ring finger protein 40, E3 ubiquitin protein ligase (Rnf40), mRNA [NM_153471] |
| LOC685621 | 0.022 | down | 2.276 | RIKEN cDNA 4933427D06 gene [Source:MGI Symbol;Acc:MGI:3026922] [ENSRNOT00000039058] |
| Pgm3 | 0.020 | down | 2.275 | Rattus norvegicus phosphoglucomutase 3 (Pgm3), mRNA [NM_001108772] |
| Wdr13 | 0.016 | down | 2.275 | Rattus norvegicus WD repeat domain 13 (Wdr13), mRNA [NM_001108247] |
| Cyp2d3 | 0.029 | down | 2.274 | Rattus norvegicus cytochrome P450, family 2, subfamily d, polypeptide 3 (Cyp2d3), mRNA [NM_173093] |
| Plekhg1 | 0.016 | down | 2.272 | Rattus norvegicus pleckstrin homology domain containing, family G (with RhoGef domain) member 1 (Plekhg1), mRNA [NM_001190999] |
| Ptx4 | 0.030 | down | 2.268 | PREDICTED: Rattus norvegicus pentraxin 4, long (Ptx4), mRNA [XM_001059245] |
| Fgfbp3 | 0.029 | down | 2.267 | Rattus norvegicus fibroblast growth factor binding protein 3 (Fgfbp3), mRNA [NM_001109165] |
| LOC102551050 | 0.019 | down | 2.257 | PREDICTED: Rattus norvegicus disks large homolog 5-like (LOC102551050), transcript variant X1, mRNA [XM_006242825] |
| RGD1565498 | 0.030 | down | 2.254 | Rattus norvegicus similar to Hypothetical protein LOC270802 (RGD1565498), transcript variant 1, mRNA [NM_001109287] |
| LOC679219 | 0.046 | down | 2.249 | PREDICTED: Rattus norvegicus ataxin-2 homolog (LOC679219), mRNA [XM_006227485] |
| Gstm6l | 0.016 | down | 2.248 | Rattus norvegicus glutathione S-transferase, mu 6-like (Gstm6l), mRNA [NM_001106464] |
| Clec14a | 0.013 | down | 2.242 | Rattus norvegicus C-type lectin domain family 14, member A (Clec14a), mRNA [NM_001014077] |
| Hebp1 | 0.013 | down | 2.238 | Rattus norvegicus heme binding protein 1 (Hebp1), mRNA [NM_001108651] |
| Csmd3 | 0.014 | down | 2.238 | PREDICTED: Rattus norvegicus CUB and Sushi multiple domains 3 (Csmd3), transcript variant X4, mRNA [XM_001063221] |
| RatNP-3b | 0.030 | down | 2.231 | Rattus norvegicus defensin RatNP-3 precursor (RatNP-3b), mRNA [NM_001079898] |
| **Nkain4** | **0.047** | **down** | **2.230** | **Rattus norvegicus Na+/K+ transporting ATPase interacting 4 (Nkain4), mRNA [NM_001106550]** |
| LOC102551453 | 0.018 | down | 2.226 | PREDICTED: Rattus norvegicus keratin, type II cytoskeletal 5-like (LOC102551453), mRNA [XM_006242450] |
| Rec8 | 0.032 | down | 2.224 | Rattus norvegicus REC8 meiotic recombination protein (Rec8), mRNA [NM_001011916] |
| Mdga1 | 0.023 | down | 2.209 | Rattus norvegicus MAM domain containing glycosylphosphatidylinositol anchor 1 (Mdga1), mRNA [NM_001107618] |
| Snx29 | 0.036 | down | 2.208 | Rattus norvegicus sorting nexin 29 (Snx29), mRNA [NM_001109526] |
| Acoxl | 0.025 | down | 2.208 | Rattus norvegicus acyl-CoA oxidase-like (Acoxl), mRNA [NM_001106508] |
| LOC691277 | 0.039 | down | 2.207 | Rattus norvegicus similar to Robo-1 (LOC691277), mRNA [NM_001109633] |
| Ube2l6 | 0.013 | down | 2.203 | Rattus norvegicus ubiquitin-conjugating enzyme E2L 6 (Ube2l6), mRNA [NM_001024755] |
| Olr1457 | 0.025 | down | 2.203 | Rattus norvegicus olfactory receptor 1457 (Olr1457), mRNA [NM_001000526] |
| Tomm20l | 0.028 | down | 2.202 | PREDICTED: Rattus norvegicus translocase of outer mitochondrial membrane 20 homolog (yeast)-like (Tomm20l), transcript variant X1, mRNA [XM_001072851] |
| Gem | 0.018 | down | 2.199 | Rattus norvegicus GTP binding protein overexpressed in skeletal muscle (Gem), mRNA [NM_001106637] |
| LOC207127 | 0.038 | down | 2.198 | Rattus norvegicus unknown protein mRNA, partial cds, [U89745] |
| Tssk4 | 0.047 | down | 2.198 | Rattus norvegicus testis-specific serine kinase 4 (Tssk4), transcript variant 1, mRNA [NM_001271325] |
| Irf7 | 0.023 | down | 2.198 | Rattus norvegicus interferon regulatory factor 7 (Irf7), mRNA [NM_001033691] |
| Tusc5 | 0.041 | down | 2.197 | Rattus norvegicus tumor suppressor candidate 5 (Tusc5), mRNA [NM_001039163] |
| Spata6l | 0.029 | down | 2.192 | Rattus norvegicus spermatogenesis associated 6-like (Spata6l), mRNA [NM_001014165] |
| Nkx6-2 | 0.022 | down | 2.189 | Rattus norvegicus NK6 homeobox 2 (Nkx6-2), mRNA [NM_001107558] |
| Olr1347 | 0.023 | down | 2.186 | Rattus norvegicus olfactory receptor 1347 (Olr1347), mRNA [NM_001001121] |
| Ovol1 | 0.021 | down | 2.185 | Rattus norvegicus ovo-like zinc finger 1 (Ovol1), mRNA [NM_001107572] |
| **Kcnc4** | **0.019** | **down** | **2.185** | **Rattus norvegicus potassium voltage gated channel, Shaw-related subfamily, member 4 (Kcnc4), mRNA [NM_001122776]** |
| Cdkl2 | 0.041 | down | 2.181 | Rattus norvegicus cyclin-dependent kinase-like 2 (CDC2-related kinase) (Cdkl2), mRNA [NM_001012035] |
| Olfm2 | 0.029 | down | 2.180 | Rattus norvegicus olfactomedin 2 (Olfm2), mRNA [NM_001015017] |
| Vav3 | 0.013 | down | 2.180 | Rattus norvegicus vav 3 guanine nucleotide exchange factor (Vav3), mRNA [NM_001191714] |
| Synm | 0.021 | down | 2.179 | Rattus norvegicus synemin, intermediate filament protein (Synm), mRNA [NM_001134858] |
| Hoxd1 | 0.018 | down | 2.177 | Rattus norvegicus homeo box D1 (Hoxd1), mRNA [NM_001105884] |
| Awat1 | 0.041 | down | 2.174 | Rattus norvegicus acyl-CoA wax alcohol acyltransferase 1 (Awat1), mRNA [NM_001109371] |
| Gtsf1l | 0.032 | down | 2.171 | PREDICTED: Rattus norvegicus gametocyte specific factor 1-like (Gtsf1l), mRNA [XM_006224737] |
| Slc7a1 | 0.013 | down | 2.167 | Rattus norvegicus solute carrier family 7 (cationic amino acid transporter, y+ system), member 1 (Slc7a1), mRNA [NM_013111] |
| Olr1564 | 0.016 | down | 2.165 | Rattus norvegicus olfactory receptor 1564 (Olr1564), mRNA [NM_001000045] |
| Tmc7 | 0.040 | down | 2.165 | Rattus norvegicus transmembrane channel-like 7 (Tmc7), mRNA [NM_001191950] |
| Atxn2l | 0.030 | down | 2.165 | Rattus norvegicus ataxin 2-like (Atxn2l), mRNA [NM_001130097] |
| Pik3c2b | 0.032 | down | 2.162 | Rattus norvegicus phosphatidylinositol-4-phosphate 3-kinase, catalytic subunit type 2 beta (Pik3c2b), mRNA [NM_001105951] |
| RGD1561795 | 0.018 | down | 2.160 | Rattus norvegicus similar to RIKEN cDNA 1700012B09 (RGD1561795), mRNA [NM_001109289] |
| LOC499573 | 0.038 | down | 2.159 | Rattus norvegicus LRRGT00056 (LOC499573), mRNA [NM_001047947] |
| Entpd2 | 0.023 | down | 2.159 | Rattus norvegicus ectonucleoside triphosphate diphosphohydrolase 2 (Entpd2), mRNA [NM_172030] |
| Xcr1 | 0.041 | down | 2.158 | Rattus norvegicus chemokine (C motif) receptor 1 (Xcr1), mRNA [NM_001106871] |
| Slc35e1 | 0.018 | down | 2.155 | Rattus norvegicus solute carrier family 35, member E1 (Slc35e1), mRNA [NM_001109107] |
| Stradb | 0.031 | down | 2.154 | Rattus norvegicus STE20-related kinase adaptor beta (Stradb), mRNA [NM_001109307] |
| Mrap | 0.043 | down | 2.153 | Rattus norvegicus melanocortin 2 receptor accessory protein (Mrap), mRNA [NM_001135834] |
| Rcor2 | 0.036 | down | 2.152 | Rattus norvegicus REST corepressor 2 (Rcor2), mRNA [NM_001013994] |
| RGD1559731 | 0.028 | down | 2.149 | PREDICTED: Rattus norvegicus similar to RIKEN cDNA 4930578C19 (RGD1559731), mRNA [XM_001057862] |
| Rnase1l1 | 0.018 | down | 2.148 | Rattus norvegicus ribonuclease, RNase A family, 1-like 1 (pancreatic) (Rnase1l1), mRNA [NM_001013232] |
| Wasf2 | 0.039 | down | 2.148 | Rattus norvegicus WAS protein family, member 2 (Wasf2), mRNA [NM_001013167] |
| Syt9 | 0.023 | down | 2.146 | Rattus norvegicus synaptotagmin IX (Syt9), mRNA [NM_053324] |
| Brwd3 | 0.027 | down | 2.145 | bromodomain and WD repeat domain containing 3 [Source:MGI Symbol;Acc:MGI:3029414] [ENSRNOT00000050336] |
| Snca | 0.016 | down | 2.144 | PREDICTED: Rattus norvegicus synuclein, alpha (non A4 component of amyloid precursor) (Snca), transcript variant X1, mRNA [XM_006236591] |
| Thsd4 | 0.022 | down | 2.142 | PREDICTED: Rattus norvegicus thrombospondin, type I, domain containing 4 (Thsd4), transcript variant X2, mRNA [XM_006226422] |
| Gdf5 | 0.026 | down | 2.141 | Protein Gdf5 [Source:UniProtKB/TrEMBL;Acc:M0R3K5] [ENSRNOT00000073736] |
| **Camk1g** | **0.036** | **down** | **2.140** | **Rattus norvegicus calcium/calmodulin-dependent protein kinase IG (Camk1g), mRNA [NM_182842]** |
| Serpina1 | 0.038 | down | 2.138 | Rattus norvegicus serpin peptidase inhibitor, clade A (alpha-1 antiproteinase, antitrypsin), member 1 (Serpina1), mRNA [NM_022519] |
| LOC361016 | 0.043 | down | 2.137 | Rattus norvegicus similar to RIKEN cDNA 4933406L09, mRNA (cDNA clone IMAGE:7301570), complete cds, [BC086559] |
| Slc28a2 | 0.030 | down | 2.135 | Rattus norvegicus solute carrier family 28 (sodium-coupled nucleoside transporter), member 2 (Slc28a2), mRNA [NM_031664] |
| Tepp | 0.030 | down | 2.135 | Rattus norvegicus testis, prostate and placenta expressed (Tepp), mRNA [NM_201655] |
| Sost | 0.037 | down | 2.135 | Rattus norvegicus sclerostin (Sost), mRNA [NM_030584] |
| Alox15b | 0.021 | down | 2.133 | Rattus norvegicus arachidonate 15-lipoxygenase, type B (Alox15b), mRNA [NM_153301] |
| Frmd4a | 0.038 | down | 2.128 | Rattus norvegicus FERM domain containing 4A (Frmd4a), mRNA [NM_001191821] |
| RGD1565057 | 0.042 | down | 2.124 | PREDICTED: Rattus norvegicus tektin-5-like (RGD1565057), transcript variant X1, mRNA [XM_006223680] |
| Sash1 | 0.032 | down | 2.121 | Protein RGD1566017 [Source:UniProtKB/TrEMBL;Acc:F1LU97] [ENSRNOT00000017688] |
| Bag4 | 0.013 | down | 2.120 | Rattus norvegicus BCL2-associated athanogene 4 (Bag4), mRNA [NM_001025130] |
| LOC100363248 | 0.029 | down | 2.117 | SOGA family member 3 [Source:MGI Symbol;Acc:MGI:1914662] [ENSRNOT00000016712] |
| Npffr1 | 0.023 | down | 2.116 | Rattus norvegicus neuropeptide FF receptor 1 (Npffr1), mRNA [NM_022291] |
| Chrm2 | 0.035 | down | 2.112 | Rattus norvegicus cholinergic receptor, muscarinic 2 (Chrm2), mRNA [NM_031016] |
| LOC303566 | 0.029 | down | 2.112 | Rattus norvegicus E2F1-inducible gene (LOC303566), mRNA [NM_001017988] |
| Ca1 | 0.021 | down | 2.112 | Rattus norvegicus carbonic anhydrase I (Ca1), mRNA [NM_001107660] |
| Lpar5 | 0.041 | down | 2.111 | PREDICTED: Rattus norvegicus lysophosphatidic acid receptor 5 (Lpar5), transcript variant X4, mRNA [XM_006225042] |
| Tmem104 | 0.016 | down | 2.109 | Rattus norvegicus transmembrane protein 104 (Tmem104), mRNA [NM_001191655] |
| Tmem52b | 0.040 | down | 2.109 | Rattus norvegicus transmembrane protein 52B (Tmem52b), mRNA [NM_001109560] |
| Pnkd | 0.046 | down | 2.108 | Rattus norvegicus paroxysmal nonkinesigenic dyskinesia (Pnkd), transcript variant 3, mRNA [NM_001134753] |
| Ccdc96 | 0.027 | down | 2.107 | PREDICTED: Rattus norvegicus coiled-coil domain containing 96 (Ccdc96), transcript variant 2, mRNA [XM_223518] |
| LOC367436 | 0.042 | down | 2.105 | Rattus norvegicus similar to Y-LINKED TESTIS-SPECIFIC PROTEIN (LOC367436), mRNA [XM_346138] |
| Slc26a11 | 0.018 | down | 2.104 | solute carrier family 26, member 11 [Source:MGI Symbol;Acc:MGI:2444589] [ENSRNOT00000054987] |
| Egfl7 | 0.022 | down | 2.103 | Rattus norvegicus EGF-like-domain, multiple 7 (Egfl7), mRNA [NM_139104] |
| RGD1559578 | 0.036 | down | 2.101 | Rattus norvegicus RGD1559578 (RGD1559578), mRNA [NM_001134497] |
| Myo3b | 0.018 | down | 2.100 | Rattus norvegicus myosin IIIB (Myo3b), mRNA [NM_001191901] |
| Grin1 | 0.037 | down | 2.099 | Rattus norvegicus glutamate receptor, ionotropic, N-methyl D-aspartate 1 (Grin1), transcript variant 2, mRNA [NM_017010] |
| Cir1 | 0.043 | down | 2.098 | Rattus norvegicus corepressor interacting with RBPJ, 1 (Cir1), mRNA [NM_001007799] |
| Mcpt1l4 | 0.047 | down | 2.098 | PREDICTED: Rattus norvegicus mast cell protease 1-like 4 (Mcpt1l4), mRNA [XM_003752801] |
| Rab3il1 | 0.028 | down | 2.095 | Rattus norvegicus RAB3A interacting protein (rabin3)-like 1 (Rab3il1), mRNA [NM_134411] |
| Aldh5a1 | 0.022 | down | 2.093 | Rattus norvegicus aldehyde dehydrogenase 5 family, member A1 (Aldh5a1), mRNA [NM_022851] |
| Epn2 | 0.020 | down | 2.092 | Rattus norvegicus epsin 2 (Epn2), transcript variant 2, mRNA [NM_001033914] |
| Aldh3a1 | 0.019 | down | 2.089 | Rattus norvegicus aldehyde dehydrogenase 3 family, member A1 (Aldh3a1), mRNA [NM_031972] |
| Tmem231 | 0.013 | down | 2.087 | Rattus norvegicus transmembrane protein 231 (Tmem231), mRNA [NM_001271031] |
| Lpo | 0.029 | down | 2.087 | Rattus norvegicus lactoperoxidase (Lpo), mRNA [NM_001105829] |
| Plek2 | 0.025 | down | 2.084 | Rattus norvegicus pleckstrin 2 (Plek2), mRNA [NM_001114180] |
| LOC102556632 | 0.016 | down | 2.081 | PREDICTED: Rattus norvegicus disks large homolog 5-like (LOC102556632), mRNA [XM_006227214] |
| RGD1565819 | 0.022 | down | 2.078 | Rattus norvegicus similar to C20orf174 (RGD1565819), mRNA [NM_001171096] |
| Frg2 | 0.018 | down | 2.077 | PREDICTED: Rattus norvegicus FSHD region gene 2 (Frg2), mRNA [XM_006221826] |
| Tmem119 | 0.035 | down | 2.076 | Rattus norvegicus transmembrane protein 119 (Tmem119), mRNA [NM_001107155] |
| RGD1562638 | 0.040 | down | 2.076 | Rattus norvegicus similar to MAP/microtubule affinity-regulating kinase 3 (RGD1562638), mRNA [NM_001100944] |
| Rtn4r | 0.046 | down | 2.075 | Rattus norvegicus reticulon 4 receptor (Rtn4r), mRNA [NM_053613] |
| Spata31d1d | 0.029 | down | 2.074 | Rattus norvegicus spermatogenesis associated 31 subfamily D, member 1D (Spata31d1d), mRNA [NM_001134606] |
| Gypc | 0.028 | down | 2.072 | Rattus norvegicus glycophorin C (Gerbich blood group) (Gypc), mRNA [NM_001013233] |
| Prickle4 | 0.023 | down | 2.072 | PREDICTED: Rattus norvegicus prickle homolog 4 (Drosophila) (Prickle4), mRNA [XM_006226724] |
| Isg15 | 0.039 | down | 2.071 | Rattus norvegicus ISG15 ubiquitin-like modifier (Isg15), mRNA [NM_001106700] |
| Ngp | 0.014 | down | 2.070 | Rattus norvegicus neutrophilic granule protein (Ngp), mRNA [NM_001106862] |
| LOC100912563 | 0.036 | down | 2.068 | PREDICTED: Rattus norvegicus insulinoma-associated protein 1-like (LOC100912563), mRNA [XM_006224680] |
| Pdzd4 | 0.020 | down | 2.066 | Rattus norvegicus PDZ domain containing 4 (Pdzd4), mRNA [NM_001135836] |
| Fam186b | 0.035 | down | 2.065 | Rattus norvegicus family with sequence similarity 186, member B (Fam186b), mRNA [NM_001134633] |
| Olr1531 | 0.047 | down | 2.062 | Rattus norvegicus olfactory receptor 1531 (Olr1531), mRNA [NM_001001102] |
| Shc1 | 0.021 | down | 2.061 | Rattus norvegicus SHC (Src homology 2 domain containing) transforming protein 1 (Shc1), transcript variant 1, mRNA [NM_001164060] |
| Flt1 | 0.015 | down | 2.059 | Rattus norvegicus FMS-related tyrosine kinase 1 (Flt1), mRNA [NM_019306] |
| Cpt1c | 0.020 | down | 2.059 | Rattus norvegicus carnitine palmitoyltransferase 1c (Cpt1c), mRNA [NM_001034925] |
| Supt4h1 | 0.026 | down | 2.058 | Rattus norvegicus suppressor of Ty 4 homolog 1 (S, cerevisiae) (Supt4h1), mRNA [NM_001105828] |
| Gstm2 | 0.018 | down | 2.058 | Rattus norvegicus glutathione S-transferase mu 2 (Gstm2), mRNA [NM_177426] |
| Pxdn | 0.046 | down | 2.058 | Rattus norvegicus peroxidasin homolog (Drosophila) (Pxdn), mRNA [NM_001271261] |
| Spink14 | 0.022 | down | 2.057 | Rattus norvegicus serine peptidase inhibitor, Kazal type 14 (Spink14), mRNA [NM_001008875] |
| RGD1306704 | 0.026 | down | 2.053 | PREDICTED: Rattus norvegicus hypothetical LOC295483 (RGD1306704), mRNA [XM_006232379] |
| RGD1563049 | 0.021 | down | 2.049 | PREDICTED: Rattus norvegicus RGD1563049 (RGD1563049), misc_RNA [XR_146001] |
| Hemgn | 0.020 | down | 2.047 | Rattus norvegicus hemogen (Hemgn), mRNA [NM_133294] |
| Syde1 | 0.043 | down | 2.044 | PREDICTED: Rattus norvegicus synapse defective 1, Rho GTPase, homolog 1 (C, elegans) (Syde1), transcript variant X1, mRNA [XM_006241068] |
| Grid2 | 0.032 | down | 2.044 | Rattus norvegicus glutamate receptor, ionotropic, delta 2 (Grid2), mRNA [NM_024379] |
| Praf2 | 0.018 | down | 2.041 | Rattus norvegicus PRA1 domain family, member 2 (Praf2), mRNA [NM_001109013] |
| RSA-14-44 | 0.022 | down | 2.040 | Rattus norvegicus RSA-14-44 protein (RSA-14-44), mRNA [NM_182670] |
| Tex11 | 0.013 | down | 2.037 | testis expressed gene 11 [Source:MGI Symbol;Acc:MGI:1933237] [ENSRNOT00000041731] |
| Htr7 | 0.029 | down | 2.037 | Rattus norvegicus 5-hydroxytryptamine (serotonin) receptor 7, adenylate cyclase-coupled (Htr7), mRNA [NM_022938] |
| Fam160b2 | 0.020 | down | 2.034 | Rattus norvegicus family with sequence similarity 160, member B2 (Fam160b2), mRNA [NM_001170474] |
| Dlc1 | 0.036 | down | 2.034 | Rattus norvegicus deleted in liver cancer 1 (Dlc1), mRNA [NM_001127446] |
| Scarf2 | 0.035 | down | 2.031 | Rattus norvegicus scavenger receptor class F, member 2 (Scarf2), mRNA [NM_001105864] |
| Ankrd23 | 0.022 | down | 2.028 | Rattus norvegicus ankyrin repeat domain 23 (Ankrd23), mRNA [NM_001108211] |
| Oprd1 | 0.022 | down | 2.027 | Rattus norvegicus opioid receptor, delta 1 (Oprd1), mRNA [NM_012617] |
| Rarres1 | 0.032 | down | 2.025 | Rattus norvegicus retinoic acid receptor responder (tazarotene induced) 1 (Rarres1), mRNA [NM_001014790] |
| Tdpoz1 | 0.019 | down | 2.021 | PREDICTED: Rattus norvegicus TD and POZ domain containing 1 (Tdpoz1), mRNA [XM_345239] |
| RGD1560314 | 0.043 | down | 2.020 | PREDICTED: Rattus norvegicus RGD1560314 (RGD1560314), mRNA [XM_006225962] |
| Olr717 | 0.047 | down | 2.018 | Rattus norvegicus olfactory receptor 717 (Olr717), mRNA [NM_001000621] |
| Fhdc1 | 0.021 | down | 2.016 | Rattus norvegicus FH2 domain containing 1 (Fhdc1), mRNA [NM_001106437] |
| Nlgn3 | 0.018 | down | 2.014 | Rattus norvegicus neuroligin 3 (Nlgn3), mRNA [NM_134336] |
| Wfikkn2 | 0.018 | down | 2.014 | PREDICTED: Rattus norvegicus WAP, follistatin/kazal, immunoglobulin, kunitz and netrin domain containing 2 (Wfikkn2), transcript variant X2, mRNA [XM_220855] |
| LOC102556288 | 0.019 | down | 2.011 | PREDICTED: Rattus norvegicus putative ZP domain-containing protein LOC729800-like (LOC102556288), mRNA [XM_006223695] |
| Olr1768 | 0.022 | down | 2.010 | Rattus norvegicus olfactory receptor 1768 (Olr1768), mRNA [NM_001000264] |
| Kif12 | 0.018 | down | 2.006 | Rattus norvegicus kinesin family member 12 (Kif12), mRNA [NM_001012102] |
| Serpinb3a | 0.044 | down | 2.005 | PREDICTED: Rattus norvegicus serine (or cysteine) peptidase inhibitor, clade B (ovalbumin), member 3A (Serpinb3a), mRNA [XM_006221436] |
| Abhd4 | 0.018 | down | 2.005 | Rattus norvegicus abhydrolase domain containing 4 (Abhd4), mRNA [NM_001108866] |
| Olr838 | 0.047 | down | 2.005 | Rattus norvegicus olfactory receptor 838 (Olr838), mRNA [NM_001000581] |
| Fam111a | 0.021 | down | 2.003 | Rattus norvegicus family with sequence similarity 111, member A (Fam111a), mRNA [NM_001109163] |
| Schip1 | 0.041 | down | 2.003 | Rattus norvegicus schwannomin interacting protein 1 (Schip1), mRNA [NM_001100666] |
| Srcin1 | 0.029 | down | 2.003 | Rattus norvegicus SRC kinase signaling inhibitor 1 (Srcin1), mRNA [NM_019378] |
| Ybx2 | 0.023 | down | 2.001 | Protein Ybx2 [Source:UniProtKB/TrEMBL;Acc:D3ZLV3] [ENSRNOT00000023003] |
| Gpr133 | 0.014 | down | 2.001 | G protein-coupled receptor 133 [Source:MGI Symbol;Acc:MGI:3041203] [ENSRNOT00000058586] |
