# Supplementary material for: Oral administration of oat beta-glucan preparations of different molecular weight results in regulation of genes connected with immune response in peripheral blood of rats with LPS-induced enteritis
Source: Eur J Nutr. 2018 Oct 4;58(7):2859–73. doi: 10.1007/s00394-018-1838-3 (PMC6769091; doi:10.1007/s00394-018-1838-3)
Supplement: Supplementary file 3 — Supplementary material 3 (DOCX 30 KB) [file 394_2018_1838_MOESM3_ESM.docx]

**Supplementary Table 3.** List of genes differentially expressed in peripheral blood of rats intravenously injected with LPS, with or without dietary sumpplementation with G2 beta-glucan (LPS-G2 vs. LPS-G0). The list presents genes whose expression was significantly changed (p <0.05 and fold change (FC) >2). Genes described in discussion are bolded.

| **GeneSymbol** | **p (Corr)** | **Regulation** | **FC (abs)** | **Description** |
| --- | --- | --- | --- | --- |
| Prodh | 0.002 | up | 8.065 | Rattus norvegicus proline dehydrogenase (oxidase) 1 (Prodh), nuclear gene encoding mitochondrial protein, mRNA [NM_001135778] |
| Tfpi | 0.049 | up | 4.585 | Rattus norvegicus tissue factor pathway inhibitor (lipoprotein-associated coagulation inhibitor) (Tfpi), transcript variant 2, mRNA [NM_001177321] |
| Edc3 | 0.040 | up | 3.808 | PREDICTED: Rattus norvegicus enhancer of mRNA decapping 3 (Edc3), transcript variant 1, mRNA [XM_001072079] |
| Gas7 | 0.021 | up | 3.379 | Rattus norvegicus mRNA for GAS-7 protein, [AJ131902] |
| **Mtus1** | **0.035** | **up** | **3.259** | **Rattus norvegicus microtubule associated tumor suppressor 1 (Mtus1), mRNA [NM_178093]** |
| Fam134a | 0.029 | up | 3.166 | Rattus norvegicus family with sequence similarity 134, member A (Fam134a), mRNA [NM_001100760] |
| Spg20 | 0.032 | up | 3.077 | Rattus norvegicus spastic paraplegia 20 (Troyer syndrome) (Spg20), mRNA [NM_001106433] |
| Cnpy3 | 0.021 | up | 2.990 | Rattus norvegicus canopy FGF signaling regulator 3 (Cnpy3), mRNA [NM_001134710] |
| Pnkd | 0.018 | up | 2.909 | Rattus norvegicus paroxysmal nonkinesigenic dyskinesia (Pnkd), transcript variant 2, mRNA [NM_001134751] |
| Usp6nl | 0.023 | up | 2.835 | Rattus norvegicus USP6 N-terminal like (Usp6nl), mRNA [NM_001106120] |
| LOC500354 | 0.018 | up | 2.651 | Rattus norvegicus similar to C030030A07Rik protein (LOC500354), mRNA [NM_001037797] |
| RGD1563145 | 0.047 | up | 2.584 | PREDICTED: Rattus norvegicus 60S ribosomal protein L13-like (RGD1563145), mRNA [XM_575281] |
| She | 0.033 | up | 2.538 | PREDICTED: Rattus norvegicus Src homology 2 domain containing E (She), mRNA [XM_001062249] |
| Ddx25 | 0.042 | up | 2.517 | Rattus norvegicus DEAD (Asp-Glu-Ala-Asp) box helicase 25 (Ddx25), mRNA [NM_031630] |
| Ethe1 | 0.025 | up | 2.476 | Rattus norvegicus ethylmalonic encephalopathy 1 (Ethe1), mRNA [NM_001106234] |
| Chrna10 | 0.047 | up | 2.460 | Rattus norvegicus cholinergic receptor, nicotinic, alpha 10 (neuronal) (Chrna10), mRNA [NM_022639] |
| Rgsl1 | 0.040 | up | 2.426 | PREDICTED: Rattus norvegicus regulator of G-protein signaling like 1 (Rgsl1), mRNA [XM_006250025] |
| Spetex-2F | 0.028 | up | 2.353 | Rattus norvegicus Spetex-2F protein (Spetex-2F), mRNA [NM_001009968] |
| Olr1239 | 0.029 | up | 2.349 | Rattus norvegicus olfactory receptor 1239 (Olr1239), mRNA [NM_001000811] |
| Scube1 | 0.047 | up | 2.349 | Rattus norvegicus signal peptide, CUB domain, EGF-like 1 (Scube1), mRNA [NM_001134884] |
| Nr1h5 | 0.021 | up | 2.338 | nuclear receptor subfamily 1, group H, member 5 [Source:MGI Symbol;Acc:MGI:3026618] [ENSRNOT00000036229] |
| Efr3b | 0.032 | up | 2.328 | Protein Efr3b [Source:UniProtKB/TrEMBL;Acc:F1LTW9] [ENSRNOT00000039251] |
| Eid2 | 0.029 | up | 2.309 | PREDICTED: Rattus norvegicus EP300 interacting inhibitor of differentiation 2 (Eid2), mRNA [XM_002725554] |
| Rnf208 | 0.042 | up | 2.294 | Rattus norvegicus ring finger protein 208 (Rnf208), mRNA [NM_001109195] |
| Uvssa | 0.021 | up | 2.273 | Rattus norvegicus UV-stimulated scaffold protein A (Uvssa), mRNA [NM_001134558] |
| Zbtb11 | 0.033 | up | 2.254 | Rattus norvegicus zinc finger and BTB domain containing 11 (Zbtb11), mRNA [NM_001107097] |
| Ear1 | 0.028 | up | 2.242 | PREDICTED: Rattus norvegicus eosinophil-associated, ribonuclease A family, member 1 (Ear1), mRNA [XM_002725056] |
| Smyd4 | 0.025 | up | 2.224 | Rattus norvegicus SET and MYND domain containing 4 (Smyd4), mRNA [NM_001105810] |
| Lysmd4 | 0.018 | up | 2.222 | PREDICTED: Rattus norvegicus LysM, putative peptidoglycan-binding, domain containing 4 (Lysmd4), transcript variant X3, mRNA [XM_006223345] |
| Fam83c | 0.028 | up | 2.187 | PREDICTED: Rattus norvegicus family with sequence similarity 83, member C (Fam83c), mRNA [XM_001066240] |
| Mroh7 | 0.029 | up | 2.182 | Rattus norvegicus maestro heat-like repeat family member 7 (Mroh7), mRNA [NM_001100965] |
| RGD1562339 | 0.018 | up | 2.181 | PREDICTED: Rattus norvegicus RGD1562339 (RGD1562339), transcript variant X1, mRNA [XM_579785] |
| Fut4 | 0.047 | up | 2.161 | Rattus norvegicus fucosyltransferase 4 (alpha (1,3) fucosyltransferase, myeloid-specific) (Fut4), mRNA [NM_022219] |
| Usp22 | 0.047 | up | 2.158 | Rattus norvegicus ubiquitin specific peptidase 22 (Usp22), mRNA [NM_001191644] |
| Prok2 | 0.023 | up | 2.153 | Rattus norvegicus prokineticin 2 (Prok2), transcript variant 1, mRNA [NM_001037541] |
| Zbtb48 | 0.031 | up | 2.150 | Rattus norvegicus zinc finger and BTB domain containing 48 (Zbtb48), mRNA [NM_001013216] |
| Rrnad1 | 0.035 | up | 2.143 | Rattus norvegicus ribosomal RNA adenine dimethylase domain containing 1 (Rrnad1), mRNA [NM_001014173] |
| Adamts15 | 0.035 | up | 2.130 | Rattus norvegicus ADAM metallopeptidase with thrombospondin type 1 motif, 15 (Adamts15), mRNA [NM_001106810] |
| Guf1 | 0.023 | up | 2.122 | Rattus norvegicus GUF1 GTPase homolog (S, cerevisiae) (Guf1), mRNA [NM_001107215] |
| Rab11fip1 | 0.027 | up | 2.119 | Rattus norvegicus RAB11 family interacting protein 1 (class I) (Rab11fip1), transcript variant 2, mRNA [NM_001197241] |
| Olr609 | 0.030 | up | 2.092 | Rattus norvegicus olfactory receptor 609 (Olr609), mRNA [NM_001000335] |
| **Il34** | **0.025** | **up** | **2.085** | **Rattus norvegicus interleukin 34 (Il34), mRNA [NM_001025766]** |
| LOC691551 | 0.041 | up | 2.084 | RIKEN cDNA 4933427G17 gene [Source:MGI Symbol;Acc:MGI:1921716] [ENSRNOT00000039447] |
| Syncrip | 0.035 | up | 2.079 | Rattus norvegicus synaptotagmin binding, cytoplasmic RNA interacting protein (Syncrip), mRNA [NM_001047916] |
| Pdk1 | 0.049 | up | 2.074 | Rattus norvegicus pyruvate dehydrogenase kinase, isozyme 1 (Pdk1), mRNA [NM_053826] |
| Sipa1l2 | 0.031 | up | 2.026 | Rattus norvegicus signal-induced proliferation-associated 1 like 2 (Sipa1l2), mRNA [NM_001009704] |
| **Atg10** | **0.042** | **up** | **2.023** | **Rattus norvegicus autophagy related 10 (Atg10), mRNA [NM_001109505]** |
| Rassf4 | 0.049 | up | 2.010 | Rattus norvegicus Ras association (RalGDS/AF-6) domain family member 4 (Rassf4), mRNA [NM_001024275] |
| Prr5l | 0.028 | up | 2.010 | Rattus norvegicus proline rich 5 like (Prr5l), mRNA [NM_001080150] |
| Ube2c | 0.043 | down | 3.550 | Rattus norvegicus ubiquitin-conjugating enzyme E2C (Ube2c), mRNA [NM_001106542] |
| Gabpb1 | 0.042 | down | 3.358 | Rattus norvegicus GA binding protein transcription factor, beta subunit 1 (Gabpb1), mRNA [NM_001039036] |
| Fam111a | 0.029 | down | 3.268 | Rattus norvegicus family with sequence similarity 111, member A (Fam111a), mRNA [NM_001109163] |
| Kcnk2 | 0.024 | down | 3.154 | Rattus norvegicus potassium channel, subfamily K, member 2 (Kcnk2), transcript variant 1, mRNA [NM_172041] |
| Spata31a5 | 0.029 | down | 3.151 | Rattus norvegicus SPATA31 subfamily A, member 5 (Spata31a5), mRNA [NM_001008359] |
| LOC690082 | 0.027 | down | 2.975 | PREDICTED: Rattus norvegicus PWWP domain-containing protein MUM1L1-like (LOC690082), transcript variant X1, mRNA [XM_003748902] |
| Olr1486 | 0.049 | down | 2.905 | Rattus norvegicus olfactory receptor 1486 (Olr1486), mRNA [NM_001000720] |
| Cst12 | 0.021 | down | 2.730 | Rattus norvegicus cystatin 12 (Cst12), mRNA [NM_153734] |
| Smim12 | 0.047 | down | 2.708 | Rattus norvegicus small integral membrane protein 12 (Smim12), mRNA [NM_001276483] |
| **Ptger3** | **0.033** | **down** | **2.696** | **Rattus norvegicus prostaglandin E receptor 3 (subtype EP3) (Ptger3), mRNA [NM_012704]** |
| RGD1563451 | 0.023 | down | 2.688 | PREDICTED: Rattus norvegicus TD and POZ domain-containing protein 2-like (RGD1563451), mRNA [XM_003753628] |
| LOC102550396 | 0.038 | down | 2.613 | Rattus norvegicus LRRGT00188 (LOC102550396), mRNA [NM_001287617] |
| LOC102551050 | 0.038 | down | 2.567 | PREDICTED: Rattus norvegicus disks large homolog 5-like (LOC102551050), transcript variant X1, mRNA [XM_006242825] |
| Mcm2 | 0.047 | down | 2.545 | Rattus norvegicus minichromosome maintenance complex component 2 (Mcm2), mRNA [NM_001107873] |
| Fam111a | 0.023 | down | 2.454 | Rattus norvegicus family with sequence similarity 111, member A (Fam111a), mRNA [NM_001109163] |
| Rnf157 | 0.018 | down | 2.437 | PREDICTED: Rattus norvegicus ring finger protein 157 (Rnf157), transcript variant X1, mRNA [XM_006220923] |
| Fam208a | 0.047 | down | 2.430 | DNA segment, Chr 14, Abbott 1 expressed [Source:MGI Symbol;Acc:MGI:1921694] [ENSRNOT00000074069] |
| Itga7 | 0.033 | down | 2.409 | Rattus norvegicus integrin, alpha 7 (Itga7), mRNA [NM_030842] |
| Parp16 | 0.018 | down | 2.402 | Rattus norvegicus poly (ADP-ribose) polymerase family, member 16 (Parp16), mRNA [NM_001014093] |
| Ccnb1 | 0.018 | down | 2.394 | Rattus norvegicus cyclin B1 (Ccnb1), mRNA [NM_171991] |
| LOC102549413 | 0.019 | down | 2.391 | PREDICTED: Rattus norvegicus uncharacterized LOC102549413 (LOC102549413), ncRNA [XR_344584] |
| LOC687869 | 0.032 | down | 2.378 | PREDICTED: Rattus norvegicus olfactory receptor 1500-like (LOC687869), partial mRNA [XM_006220725] |
| Clybl | 0.042 | down | 2.357 | Rattus norvegicus citrate lyase beta like (Clybl), mRNA [NM_001100685] |
| LOC102547119 | 0.019 | down | 2.330 | PREDICTED: Rattus norvegicus spermatogenesis-associated protein 31C2-like (LOC102547119), mRNA [XM_006257130] |
| LRRTM1 | 0.021 | down | 2.302 | Rattus norvegicus leucine rich repeat transmembrane neuronal 1 (LRRTM1), mRNA [NM_001109374] |
| Pou4f2 | 0.043 | down | 2.298 | Rattus norvegicus POU class 4 homeobox 2 (Pou4f2), mRNA [NM_134355] |
| RGD1566307 | 0.023 | down | 2.276 | PREDICTED: Rattus norvegicus leukocyte immunoglobulin-like receptor subfamily B member 3-like (RGD1566307), transcript variant X9, mRNA [XM_006223047] |
| RGD1566248 | 0.035 | down | 2.271 | PREDICTED: Rattus norvegicus necdin-like (RGD1566248), mRNA [XM_006222985] |
| RGD1564836 | 0.027 | down | 2.268 | Protein RGD1564836 [Source:UniProtKB/TrEMBL;Acc:D3ZAX1] [ENSRNOT00000038940] |
| Arpc2 | 0.023 | down | 2.268 | Rattus norvegicus actin related protein 2/3 complex, subunit 2 (Arpc2), mRNA [NM_001106919] |
| Tmem200a | 0.036 | down | 2.258 | Rattus norvegicus transmembrane protein 200A (Tmem200a), mRNA [NM_001109135] |
| Olr715 | 0.048 | down | 2.251 | Rattus norvegicus olfactory receptor 715 (Olr715), mRNA [NM_001000623] |
| Spink14 | 0.023 | down | 2.215 | Rattus norvegicus serine peptidase inhibitor, Kazal type 14 (Spink14), mRNA [NM_001008875] |
| Pnkd | 0.023 | down | 2.210 | Rattus norvegicus paroxysmal nonkinesigenic dyskinesia (Pnkd), transcript variant 3, mRNA [NM_001134753] |
| Phf11b | 0.042 | down | 2.204 | Rattus norvegicus PHD finger protein 11B (Phf11b), mRNA [NM_001014235] |
| Gzmbl3 | 0.023 | down | 2.189 | Protein Gzmbl3 [Source:UniProtKB/TrEMBL;Acc:G3V9D4] [ENSRNOT00000045209] |
| Dzip3 | 0.021 | down | 2.179 | PREDICTED: Rattus norvegicus DAZ interacting zinc finger protein 3 (Dzip3), transcript variant X2, mRNA [XM_006221122] |
| Mtss1l | 0.038 | down | 2.161 | Rattus norvegicus metastasis suppressor 1-like (Mtss1l), mRNA [NM_001191558] |
| Olr1121 | 0.028 | down | 2.157 | Rattus norvegicus olfactory receptor 1121 (Olr1121), mRNA [NM_001000516] |
| Rdh5 | 0.035 | down | 2.156 | Protein Rdh5 [Source:UniProtKB/TrEMBL;Acc:D3ZPQ8] [ENSRNOT00000010217] |
| Xrcc4 | 0.038 | down | 2.152 | Rattus norvegicus X-ray repair complementing defective repair in Chinese hamster cells 4 (Xrcc4), mRNA [NM_001006999] |
| **Kcnmb2** | **0.048** | **down** | **2.137** | **Rattus norvegicus potassium large conductance calcium-activated channel, subfamily M, beta member 2 (Kcnmb2), mRNA [NM_176861]** |
| **Kcnj12** | **0.047** | **down** | **2.091** | **Rattus norvegicus potassium inwardly-rectifying channel, subfamily J, member 12 (Kcnj12), mRNA [NM_053981]** |
| Dnajc11 | 0.033 | down | 2.068 | Rattus norvegicus DnaJ (Hsp40) homolog, subfamily C, member 11 (Dnajc11), mRNA [NM_001108694] |
| Tnn | 0.022 | down | 2.021 | Rattus norvegicus tenascin N (Tnn), mRNA [NM_001107189] |
| Cyp2b12 | 0.023 | down | 2.021 | cytochrome P450, family 2, subfamily b, polypeptide 12 (Cyp2b12), mRNA [Source:RefSeq mRNA;Acc:NM_017156] [ENSRNOT00000041580] |
| Olr1303 | 0.047 | down | 2.020 | Rattus norvegicus olfactory receptor 1303 (Olr1303), mRNA [NM_001000796] |
| Zfp385d | 0.032 | down | 2.005 | Rattus norvegicus zinc finger protein 385D (Zfp385d), mRNA [NM_001013992] |
